# Theoretical framework for a decision support system for micro-enterprise supermarket investment risk assessment using novel picture fuzzy hypersoft graph

**DOI:** 10.1371/journal.pone.0273642

**Published:** 2023-03-07

**Authors:** Muhammad Saeed, Muhammad Imran Harl, Muhammad Haris Saeed, Ibrahim Mekawy

**Affiliations:** 1 Department of Mathematics, University of Management and Technology, Lahore, Pakistan; 2 Department of Chemistry, University of Management and Technology, Lahore, Pakistan; 3 Department of Mathematics, College of Science and Arts, Qassim University, Al-Rass, Kingdom of Saudi Arabia; Xinjiang Technical Institute of Physics and Chemistry, Chinese Academy of Sciences, CHINA

## Abstract

Risk evaluation has always been of great interest for individuals wanting to invest in various businesses, especially in the marketing and product sale centres. A finely detailed evaluation of the risk factor can lead to better returns in terms of investment in a particular business. Considering this idea, this paper aims to evaluate the risk factor of investing in different nature of products in a supermarket for a better proportioning of investment based on the product’s sales. This is achieved using novel Picture fuzzy Hypersoft Graphs. Picture Fuzzy Hypersoft set (PFHSs) is employed in this technique, a hybrid structure of Picture Fuzzy set and Hypersoft Set. These structures work best for evaluating uncertainty using membership, non-membership, neutral, and multi-argument functions, making them ideal for Risk Evaluation studies. Also, the concept of the PFHS graph with the help of the PFHS set is introduced with some operations like the cartesian product, composition, union, direct product, and lexicographic product. This method presented in the paper provides new insight into product sale risk analysis with a pictorial representation of its associated factors.

## 1 Introduction

The world is evolving with humanity learning from processing immense amounts of data available in various forms, no matter how convoluted it may seem, in the hope that it may lead to some insight. Processing the data in multiple ways has allowed for a more analytic and scenario-based understanding of current affairs, allowing for effective decision-making in science and business. Keeping this notion in mind, business individuals in product sales and managing supermarkets require product sales supply and demand analytics so that the stock is always the optimum amount to go off the shelves while in the best condition.

Utilizing this information allows for the design of promotional strategies for product sales benefitting the business. Most customers in supermarkets are not subjected to confer with the prices while buying the product, so there is minimal need for them to confer with their internal reference prices, allowing marketing strategists more room to design marketing strategies for business growth. For this purpose, numerous mathematical and computational tools have been designed to analyze these marketing indicators. Among these computational methods, some methods are discussed, but among these methods, attributes are not divided into sub-attributes. To encounter this problem, this paper presents a novel concept of picture fuzzy hypersoft graphs that allows for graphical analysis of multi-criteria decision-making problems while discussing a particular application regarding investment division and risk analysis of products in a supermarket or micro-enterprise setting. The paper first explains some mathematical literature and techniques used for market risk analysis. The novelty of the picture fuzzy hypersoft graph is defined by applying division of investment and evaluating the risk of supermarket products by tackling it as a multi-criteria decision-making problem.

The concept of fuzzy graph was developed by defining a graphical relationship among fuzzy sets by Rosenfeld in 1975 [1]. With the introduction of this concept, fuzzy set extensions like intuitionistic fuzzy set (IFS) [2] were further improved to picture fuzzy set (PFS) [3]. This extension allowed for addressing opinion based data (i.e., yes, no, abstain, and refusal) by addition of a neutral membership function to the already existing IFS. The operators and basic definitions of the PFS concept have been presented in [4, 5]. PFS has been discussed in numerous application based research in diverse fields like information technology, pharmacy, operational research, and business. The above-discussed fuzzy structures fall short when it comes to addressing data where the attributes are divided into multiple sub-attributes. For this purpose, Soft Set (SS) were extended to Hypersoft Set (HSS) by Smarandache which allowed modifying the one parameter set into a multi-parameter set in the domain set of function [6]. The properties of HSS were developed by Saeed et al. (i.e., HS subset, union, intersection, etc.) and developed hybrids of this HSS structure with structures from fuzzy and neutrosophic set theory [7, 8]. Literature does revela some fuzzy hyperosft hybrid structures including Pythagorean fuzzy hypersoft set and Intuitionistic Fuzzy Hypersoft Set (IFHSS) which is a generalized version of the Intuitionistic Fuzzy Soft Set (IFSS) [9, 10]. To elaborate the versatility and operation of the hybrid fuzzy hypersoft and neutrosophic hypersoft structures, they were applied for the development of medical decision support systems, pattern recognition to monitor the spread of COVID-19 with real-time population data over the period of 1.5 years [11–18].

## 2 Literature review

The development of a fuzzy graph structure lead to the expansion of hybrid structures of Fuzzy Sets and Graph Theory (i.e., balanced interval-valued fuzzy graphs [19], fuzzy planar graphs [20, 21], m- step fuzzy competition graphs [22], fuzzy threshold graph [23], fuzzy cubic graph [24], and fuzzy k-competition graph [25]). The interval-valued fuzzy threshold graph was developed and its properties were explored by Pramanik et al. [26]. The concept of planarity was applied to a simple Bipolar Fuzzy Graph leading to an extension to the already existing structure to a more refined bipolar fuzzy planar graphs [27]. Some other hybrid extension of the Fuzzy Planar graphs include interval-valued fuzzy graph [28] and interval-valued fuzzy planar graph [29]. The concept of Fuzzy Hypergraphs was developed by Voskoglou et al. [30] alongside several other related graphical strctures. Another term called Intuitionistic fuzzy competition graph was introduced by Sahoo et al. [31] while Balanced intuitionistic fuzzy graphs were introduced by Karunambigai et al. [32]. When talking about Intuitionistic Fuzzy Graphs, Sahoo et al. applied these intricate structure to numerous real world applications [33–35].

Numerous studies related to Picture Fuzzy Graphs and their properties like edge domination have been reported in literature alongside their applications and hybrid structures [36–38]. The hybrid structures like the Picture Dombi Fuzzy Graph, interval-valued picture uncertain linguistic generalized Hamacher aggregation operators, and the q-rung picture fuzzy graph have significant applications in decision-making problems, design of decision support systems, and associating fuzzy graphical structures in topological spaces [39–43]. These decision support systems require fuzzy aggregation opertors that combine the observations recorded in the data for manipulation in a fuzzy environment [44]. Some examples of these studies include pattern recognition using novel similarity measures for T-spherical fuzzy sets, a policy design aid by using an averaging operator of T-spherical fuzzy set [45, 46]. The T-spherical fuzzy set was presented as a generalization of the previously existing Fuzzy Set concepts (i.e., Intuitionistic fuzzy set and the Picture fuzzy set) [47].

Fuzzy set theory and concepts from Graph theory have been combined for the development of hybrid structures with better ability to handle and express data to extract better results. Picture fuzzy labeling graphs were reported by Devaraj et al. in 2020 alongside its application in decision making [47, 48]. Another interesting concept of fuzzy cross-entropy for picture hesitant fuzzy sets and introduction of polarity in addressing of attributes using Bipolar soft sets were developed by Mahmood et al. for addressing attributes of different nature in decision making problems [49, 50]. A generalized concept of picture fuzzy soft set alongside its application was put forward by Khan et al. [50, 51]. Intuitionistic multiplicative set operational laws and their associated aggregation operators for processing was developed by Garg alongside its application in decision support systems [40]. Balanced Picture Fuzzy Graphs were explored in 2021 by Amanathulla et al. [51, 52]. Numerous related hybrid fuzzy graph theoretical concepts relating to different aspects of their applications are presented in the following studies [53–58].

The concept of hybrids of hypersoft structures and graph theory have been discussed by Saeed et al. including intricate concepts like the hypersoft graph, a hypersoft subgraph, a complete hypersoft graph, and a hypersoft tree etc [59]. Due to the fast and evolving nature of businesses nowadays, its essential to calculate every single move with extreme delicacy and process a plethora of information in order to assess the risk with respect to inventory management. Literature reveals some studies that involve the use of mower based on fuzzy logic approach for effective and efficient methods for customer service [60]. Similar economic-mathematical models allow for the forecasting of growth of agricultural sector of Ukraine in order to make suitable assessment for people in business and agarians [61]. These assessments allow for timely making business decision based on facts and calculations reducing the risk in terms of loss. Another approach presented in [62] aims for evaluation of enterprise based systems with multiple criteria for analysis and designing a decision-support system. An approach presented in [63] applied clique covering of a fuzzy graph for parametric optimization for development of optimal business strategies.

## 3 Motivation

Numerous methods have been reported in the literature that has been used to solve MADM problems by using concepts from Fuzzy and Soft Sets. All these methods have some restrictions, such as when elements of the attributes set contain further sub-attributes and elements of each attribute set have no relationship. In order to address these difficulties, the concept of Picture Fuzzy Hypersoft graph (PFHSG) is introduced in this paper which is a hybrid structure of the Picture Fuzzy Hypersoft set with Graph Theory. The presented structure was then employed to develop a decision-support algorithm that uses a combination of concepts form the Hypersoft and Fuzzy Set theory. For a proper explanation, some basic terminologies are defined in Section 1, section 2 focuses on the presentation of properties of the PFHSG, while section 3 highlights the multi-attribute decision support system that was designed to based on the PFHSG for addressing intricate decision-making issues in a graphical manner. In Section 4, the algorithm is numerically applied on a risk assessment problem in order to elaborate its working principle and operations. The final section 4 is presents a brief conclusion of the paper.

## 4 Preliminaries

This section focuses on definitions of IFS, PFS, SS, HS, PFSS, PFG, and HSG.

Atanassov [2] defined IFS a direct extension of the fuzzy set which consist a membership function and a non-membership function. It overcomes defects of fuzzy sets.

**Definition 1: Intuisionistic Fuzzy Set** [2]

An intuitionistic fuzzy set X on universe of discourse *X* = {*x*_1_, *x*_2_, …‥, *x*_*n*_} is an object of the form:

$$\tilde{L}=\left\{\left\langle {\ddot{\mu }}_{\tilde{L}}\left(x_{i}\right),{\ddot{\nu }}_{\tilde{L}}\left(x_{i}\right)\right\rangle |x_{i}\in X\right\}$$

where ${\ddot{\mu }}_{\tilde{L}}\left(x_{i}\right):X\to \left[0,1\right]$ is called degree of membership of *x*_*i*_ in $\tilde{L}$ ${\ddot{\nu }}_{\tilde{L}}\left(x_{i}\right):X\to \left[0,1\right]$ is called degree of non membership of *x*_*i*_ in $\tilde{L}$ and $0\leq {\ddot{\mu }}_{\tilde{L}}\left(x_{i}\right)+{\ddot{\nu }}_{\tilde{L}}\left(x_{i}\right)\leq 1$, ∀*x*_*i*_ ∈ *X* $\pi_{\tilde{L}}\left(x_{i}\right)=1-{\ddot{\mu }}_{\tilde{L}}\left(x_{i}\right)-{\ddot{\nu }}_{\tilde{L}}\left(x_{i}\right)$ is called hesitancy degree of *x*_*i*_ in $\tilde{L}$, ∀*x*_*i*_ ∈ *X*, $0\leq \pi_{\tilde{L}}\left(x_{i}\right)\leq 1$.

**Definition 2: Picture Fuzzy Set** [4]

In 2013, Coung [4] introduced PFS in order to solve inconsistent and uncertain information in real life. Picture fuzzy set consists of three functions: positive membership function, neutral membership function, and negative membership function. A good example of PFS is electoral voting. A Picture fuzzy set on universe of discourse *X* = {*x*_1_, *x*_2_, …‥, *x*_*n*_} is an object of the form:

$${\tilde{L}}_{1}=\left\{\left\langle {\ddot{\mu }}_{{\tilde{L}}_{1}}\left(x_{i}\right),{\ddot{\eta }}_{{\tilde{L}}_{1}}\left(x_{i}\right),{\ddot{\nu }}_{{\tilde{L}}_{1}}\left(x_{i}\right)\right\rangle |x_{i}\in X\right\}$$

where ${\ddot{\mu }}_{{\tilde{L}}_{1}}\left(x_{i}\right):X\to \left[0,1\right]$ is called degree of positive membership of *x*_*i*_ in ${\tilde{L}}_{1}$ where ${\ddot{\eta }}_{{\tilde{L}}_{1}}\left(x_{i}\right):X\to \left[0,1\right]$ is called degree of neutral membership of *x*_*i*_ in ${\tilde{L}}_{1}$ ${\ddot{\nu }}_{{\tilde{L}}_{1}}\left(x_{i}\right):X\to \left[0,1\right]$ is called degree of negative membership of *x*_*i*_ in ${\tilde{L}}_{1}$ and $0\leq {\ddot{\mu }}_{{\tilde{L}}_{1}}\left(x_{i}\right)+{\ddot{\eta }}_{{\tilde{L}}_{1}}\left(x_{i}\right)+{\ddot{\nu }}_{{\tilde{L}}_{1}}\left(x_{i}\right)\leq 1$, ∀*x*_*i*_ ∈ *X* $\rho_{{\tilde{L}}_{1}}\left(x_{i}\right)=1-{\ddot{\mu }}_{{\tilde{L}}_{1}}\left(x_{i}\right)-{\ddot{\eta }}_{{\tilde{L}}_{1}}\left(x_{i}\right)-{\ddot{\nu }}_{{\tilde{L}}_{1}}\left(x_{i}\right)$ is called degree of refusal membership of *x*_*i*_ in ${\tilde{L}}_{1}$, ∀*x*_*i*_ ∈ *X* The set of all picture fuzzy subsets on universe of discourse ${\tilde{L}}_{1}$ is denoted by PFSs(X).

Some basic operation of PFS are defined as follows:

**Operations in Picture Fuzzy Soft Sets** [4]

Let ${\tilde{L}}_{1}=\left\{\langle {\ddot{\mu }}_{{\tilde{L}}_{1}}\left(x\right),{\ddot{\eta }}_{{\tilde{L}}_{1}}\left(x\right),{\ddot{\nu }}_{{\tilde{L}}_{1}}\left(x\right)\rangle |x\in X\right\}$ and ${\tilde{L}}_{2}=\left\{\langle {\ddot{\mu }}_{{\tilde{L}}_{2}}\left(x\right),{\ddot{\eta }}_{{\tilde{L}}_{2}}\left(x\right),{\ddot{\nu }}_{{\tilde{L}}_{2}}\left(x\right)\rangle |x\in X\right\}$ be two PFSs on universe X. Then the operations between the sets ${\tilde{L}}_{1}$ and ${\tilde{L}}_{2}$ are defined as follows:

(i)
$\;{\tilde{L}}_{1}\subseteq {\tilde{L}}_{2}$ iff ${\ddot{\mu }}_{{\tilde{L}}_{1}}\left(x\right)\leq {\ddot{\mu }}_{{\tilde{L}}_{2}}\left(x\right)$, ${\ddot{\eta }}_{{\tilde{L}}_{1}}\left(x\right)\leq {\ddot{\eta }}_{{\tilde{L}}_{2}}\left(x\right)$ and ${\ddot{\nu }}_{{\tilde{L}}_{1}}\left(x\right)\geq {\ddot{\nu }}_{{\tilde{L}}_{2}}\left(x\right)$

${\tilde{L}}_{1}={\tilde{L}}_{2}$ iff ${\tilde{L}}_{1}\subseteq {\tilde{L}}_{2}$ and ${\tilde{L}}_{2}\subseteq {\tilde{L}}_{1}$
(ii)
$\left({\tilde{L}}_{1}\right)\cup \left({\tilde{L}}_{2}\right)$ = $\{\left(x,max\left({\ddot{\mu }}_{{\tilde{L}}_{1}}\left(x\right),{\ddot{\mu }}_{{\tilde{L}}_{2}}\left(x\right)\right)\right)$, $\left(x,min\left({\ddot{\eta }}_{{\tilde{L}}_{1}}\left(x\right),{\ddot{\eta }}_{{\tilde{L}}_{2}}\left(x\right)\right)\right),\left(x,min\left({\ddot{\nu }}_{{\tilde{L}}_{1}}\left(x\right),{\ddot{\nu }}_{{\tilde{L}}_{2}}\left(x\right)\right)\right)\}$

(iii)
${\tilde{L}}_{1}\cap {\tilde{L}}_{2}$ = $\{\left(x,min\left({\ddot{\mu }}_{{\tilde{L}}_{1}}\left(x\right),{\ddot{\mu }}_{{\tilde{L}}_{2}}\left(x\right)\right)\right)$, $\left(x,min\left({\ddot{\eta }}_{{\tilde{L}}_{1}}\left(x\right),{\ddot{\eta }}_{{\tilde{L}}_{2}}\left(x\right)\right)\right),\left(x,max\left({\ddot{\nu }}_{{\tilde{L}}_{1}}\left(x\right),{\ddot{\nu }}_{{\tilde{L}}_{2}}\left(x\right)\right)\right)\}$

(iv) let ${\tilde{L}}_{1}=\left\{\langle {\ddot{\mu }}_{{\tilde{L}}_{1}}\left(x\right),{\ddot{\eta }}_{{\tilde{L}}_{1}}\left(x\right),{\ddot{\nu }}_{{\tilde{L}}_{1}}\left(x\right)\rangle |x\in X\right\}$ then ${\tilde{L}}_{1}^{c}=\left\{\langle {\ddot{\nu }}_{{\tilde{L}}_{1}}\left(x\right),{\ddot{\eta }}_{{\tilde{L}}_{1}}\left(x\right),{\ddot{\mu }}_{{\tilde{L}}_{1}}\left(x\right)\rangle |x\in X\right\}$

**Definition 4: Soft Set** [64]

A new scientific instrument in which parameter space is finite or infinite, even if universal set is finite, is known as a soft set. It was proposed by Molodtsov [64] for parameterized uncertainty handling purposes. A mapping F:A→P(U)

(*F*, *A*) is called a soft set over U, where A is set of parameters.

**Definition 5: Picture Fuzzy Soft Set** [65]

In 2015, Yang et al [65] proposed PFSs which is combination of PFS and SS. Let E be a parametric space with a U universal set. Now, the set of all picture fuzzy sets be represented by U. In this scenario, a picture fuzzy soft set (PFSS) is a pair (*F*, *A*) where *A* ⊆ *E* and F is a mapping as shown:

F:A→PF(U).

**Definition 6: Hypersoft Set** [6]

In 2018, Smarandache [6] put forward the concept of Hypersoft Set, which is a generalization of SS by transforming the mapping into a multi-attribute. Suppose *b*_1_, *b*_2_, ……, *b*_*n*_, for *b* ≥ 1, be n distinct traits, whose corresponding trait values are respectively the sets $Q_{1},Q_{2},\ldots ‥,Q_{n}$, with $Q_{r}⋂Q_{s}=\phi$, *i* ≠ *j*, and r,s ∈ {1, 2, …., *n*}. Then the pair $\left(\ddot{\Psi },Q_{1}\times Q_{2}\times \ldots ‥\times Q_{n}\right)$, where $\ddot{\Psi }:Q_{1}\times Q_{2}\times \ldots ‥\times Q_{n}$ → P(U) is called a Hypersoft Set over U.

**Definition 6: Fuzzy Graph** [35]

A fuzzy graph G is defined by ordered paired of functions of *μ* and *ρ*, and *G* = (*μ*, *ρ*). *μ* represents a fuzzy subset of V (A finite non-empty set of vertices) and *ρ* represents a symmetric fuzzy relation on *μ*, i.e., *μ* : *V* → [0, 1] and *ρ* : *V* × *V* → [0, 1] such that:

ρ(p,q)≤min(μ(p),ρ(q)),∀p,q∈V.

**Definition 7: Complete Fuzzy Graph** [35]

A fuzzy graph *G* = (*μ*, *ρ*) is called complete fuzzy graph if

ρ(p,q)=min(μ(p),ρ(q)),∀p,q∈V.

**Definition 8: Strong Fuzzy Graph** [35]

A fuzzy graph *G* = (*μ*, *ρ*) is called strong fuzzy graph if

ρ(p,q)=min(μ(p),ρ(q)),∀p,q∈V.

**Definition 9: Complement of a Fuzzy Graph** [35]

The complement of a fuzzy graph *G* = (*μ*, *ρ*) is a fuzzy graph and it is represented as *G*^*c*^ = (*μ*^*c*^, *ρ*^*c*^), where *μ*^*c*^ = *μ* and

ρ(p,q)=min(μ(p),ρ(q))-ρ(p,q),∀p,q∈V.

**Definition 10: Picture Fuzzy Graph** [35]

Suppose P=(V,E) be a graph. A pair D=(S,G) be a PFG where $S=({\ddot{\mu }}_{S},{\ddot{\eta }}_{S},{\ddot{\nu }}_{S})$ is PFS on V and $G=({\ddot{\mu }}_{G},{\ddot{\eta }}_{G},{\ddot{\nu }}_{G})$ is PFS on E⊆V×V. Also, for all u,v∈E ${\ddot{\mu }}_{G}\left(u,v\right)⪯{\ddot{\mu }}_{S}\left(u\right)\wedge {\ddot{\mu }}_{S}\left(v\right),\;\;{\ddot{\mu }}_{G}\left(u,v\right)⪯{\ddot{\mu }}_{S}\left(u\right)\wedge {\ddot{\mu }}_{S}\left(v\right)$ , ${\ddot{\mu }}_{G}\left(u,v\right)⪰{\ddot{\mu }}_{S}\left(u\right)\vee {\ddot{\mu }}_{S}\left(v\right)$ .

## 5 Picture fuzzy hypersoft graph

In [65], hybrid model of a PFS and a SS is defined. Keeping that notion in mind, we introduce PFHSS as an extension of PFSS, which helps for playing a crucial role in decision-making for multi-attribute characteristics.

**Definition 11: Picture Fuzzy Hypersoft Set**

Suppose m disjoint attribute-valued sets are *p*_1_, *p*_2_, *p*_3_, …, *p*_*m*_ then their corresponding *m* distinct attributes are $P_{1},P_{2},P_{3},....,P_{m}$,respectively. And $P=P_{1}\times P_{2}\times P_{3}\times ....\times P_{m}$. A mapping is given by $\ddot{H}:P\to PF\left(U\right)$

$$\ddot{H}\left(\ddot{t}\right)=\left\{\left\langle \mu_{\ddot{H}\left(\ddot{t}\right)}\left({\ddot{j}}_{i}\right),\eta_{\ddot{H}\left(\ddot{t}\right)}\left({\ddot{j}}_{i}\right),\nu_{\ddot{H}\left(\ddot{t}\right)}\left({\ddot{j}}_{i}\right)\right\rangle |\left({\ddot{j}}_{i}\right)\in U\right\}\;for\;any\;\ddot{t}\in P$$

then pair $\left(\ddot{H},P\right)$ represent PFHSS.

**Definition 12: Picture Fuzzy Hypersoft Graph**

Suppose P=(C,S) be a graph. A pair Y=(U,W) be a PFHSG where $U=({\overset{˘}{\mu }}_{U},{\overset{˘}{\eta }}_{U},{\overset{˘}{\nu }}_{U})$ is PFHSS on C and $W=({\overset{˘}{\mu }}_{W},{\overset{˘}{\eta }}_{W},{\overset{˘}{\nu }}_{W})$ is PFHSS on S⊆U×U, for all m,n∈C ${\overset{˘}{\mu }}_{W}\left(m,n\right)⪯{\overset{˘}{\mu }}_{U}\left(m\right)\wedge {\overset{˘}{\mu }}_{U}\left(n\right)$, ${\overset{˘}{\mu }}_{W}\left(m,n\right)⪯{\overset{˘}{\mu }}_{U}\left(m\right)\wedge {\overset{˘}{\mu }}_{U}\left(n\right)$, ${\overset{˘}{\mu }}_{W}\left(m,n\right)⪰{\overset{˘}{\mu }}_{U}\left(m\right)\vee {\overset{˘}{\mu }}_{U}\left(n\right)$ and $\;\;0⪯{\overset{˘}{\mu }}_{U}+{\overset{˘}{\eta }}_{U}+{\overset{˘}{\nu }}_{U}⪯1$

**Example of Picture Fuzzy Hypersoft Graph**

Consider Y=(U,W) be a PFHSG such that $U=\{{\ddot{o}}_{1},{\ddot{o}}_{2},{\ddot{o}}_{3},{\ddot{o}}_{4}\}$ and $W=\{{\ddot{o}}_{1}{\ddot{o}}_{2},{\ddot{o}}_{1}{\ddot{o}}_{3},{\ddot{o}}_{1}{\ddot{o}}_{4},{\ddot{o}}_{2}{\ddot{o}}_{4},{\ddot{o}}_{3}{\ddot{o}}_{4}\}$. Suppose $\left(\ddot{H},\tilde{L}\right)$ be PFHSS over U. Let $U=\{{\ddot{o}}_{1},{\ddot{o}}_{2},{\ddot{o}}_{3},{\ddot{o}}_{4}\}$ be four refrigerators. Let $R=\{a_{1},a_{2},a_{3}\}$ be parameters set, where each *a*_*i*_ stands for Company, Size, and Color be the attributes values respectively, {*H*_1_, *H*_2_, *H*_3_} be attribute values against each *a*_*i*_.

Let *H*_1_ = {*b*_11_ = Company X, *b*_12_ = Company Y, *b*_13_ = Company Z }

*H*_2_ = {*b*_21_ = Grey, *b*_22_ = White, *b*_23_ = Golden}

*H*_3_ = {*b*_31_ = Small}

$$\tilde{L}=H_{1}\times H_{2}\times H_{3}$$

There are a total of 9 outcomes but for simplicity, only three outcomes have been selected for the analysis.

$$\tilde{L}=\{\begin{array}{c} \;{\ddot{t}}_{1}=\left(b_{11},b_{21},b_{31}\right),\;\;\;{\ddot{t}}_{2}=\left(b_{12},b_{31}\right),\\ {\ddot{t}}_{3}=\left(b_{12},b_{22},b_{31}\right) \end{array}\}$$

$$\left(\ddot{H},\tilde{L}\right)=\{\begin{array}{c} \;\ddot{H}\left({\ddot{t}}_{1}\right)=\{\langle 0.7,0.1,0.1\rangle /{\ddot{o}}_{1},\langle 0.3,0.2,0.4\rangle /{\ddot{o}}_{2},\\ \;\;\;\;\;\;\;\;\;\;\;\;\langle 0.1,0.5,0.3\rangle /{\ddot{o}}_{3},\langle 0.4,0.1,0.3\rangle /{\ddot{o}}_{4}\},\\ \ddot{H}\left({\ddot{t}}_{2}\right)=\{\langle 0.6,0.1,0.2\rangle /{\ddot{o}}_{1},\langle 0.4,0.1,0.3\rangle /{\ddot{o}}_{2},\\ \;\;\;\;\;\;\;\;\;\;\;\;\langle 0.7,0.1,0.1\rangle /{\ddot{o}}_{3},\langle 0.2,0.5,0.2\rangle /{\ddot{o}}_{4},\},\\ \ddot{H}\left({\ddot{t}}_{3}\right)=\{\langle 0.2,0.3,0.5\rangle /{\ddot{o}}_{1},\langle 0.1,0.1,0.6\rangle /{\ddot{o}}_{2},\\ \;\;\;\;\;\;\;\;\;\;\;\;\langle 0.2,0.1,0.7\rangle /{\ddot{o}}_{3},\langle 0.8,0.1,0.1\rangle /{\ddot{o}}_{4}\} \end{array}\}$$

The vertices set of PFHSG presented in Table 1.

**Table 1:** Vertices set of PFHSG.

U	${\ddot{t}}_{1}$	${\ddot{t}}_{2}$	${\ddot{t}}_{3}$
${\ddot{o}}_{1}$	〈0.7, 0.1, 0.1〉	〈0.6, 0.1, 0.2〉	〈0.2, 0.3, 0.5〉
${\ddot{o}}_{2}$	〈0.3, 0.2, 0.4〉	〈0.4, 0.1, 0.3〉	〈0.1, 0.1, 0.6〉
${\ddot{o}}_{3}$	〈0.1, 0.5, 0.3〉	〈0.7, 0.1, 0.1〉	〈0.2, 0.1, 0.7〉
${\ddot{o}}_{4}$	〈0.4, 0.1, 0.3〉	〈0.2, 0.5, 0.2〉	〈0.8, 0.1, 0.1〉

**Fig 1:**
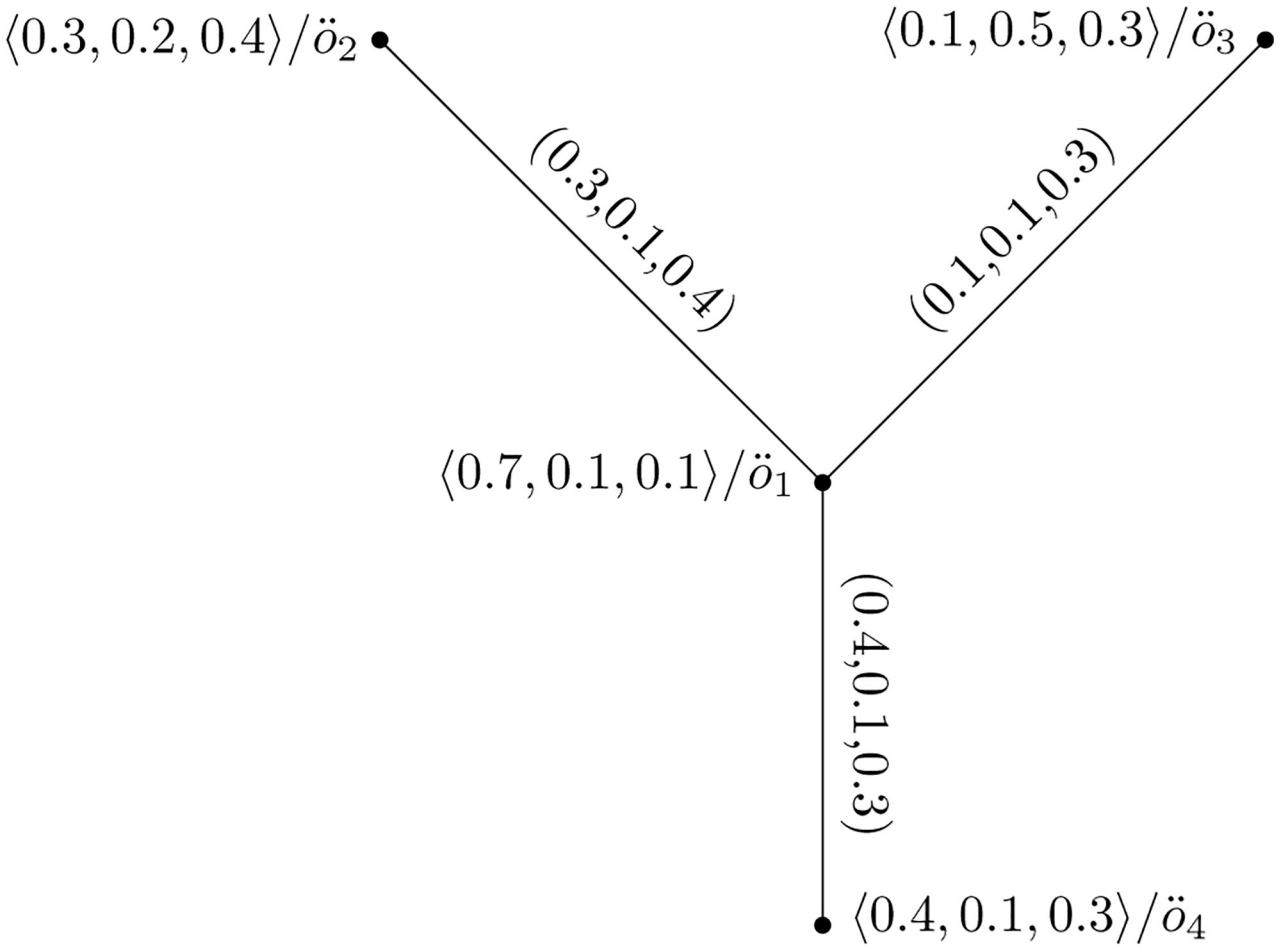
Picture fuzzy hypersoft graph $Y_{1}=\left(\ddot{H}\left({\ddot{t}}_{1}\right),\ddot{G}\left({\ddot{t}}_{1}\right)\right)$.

**Fig 2:**
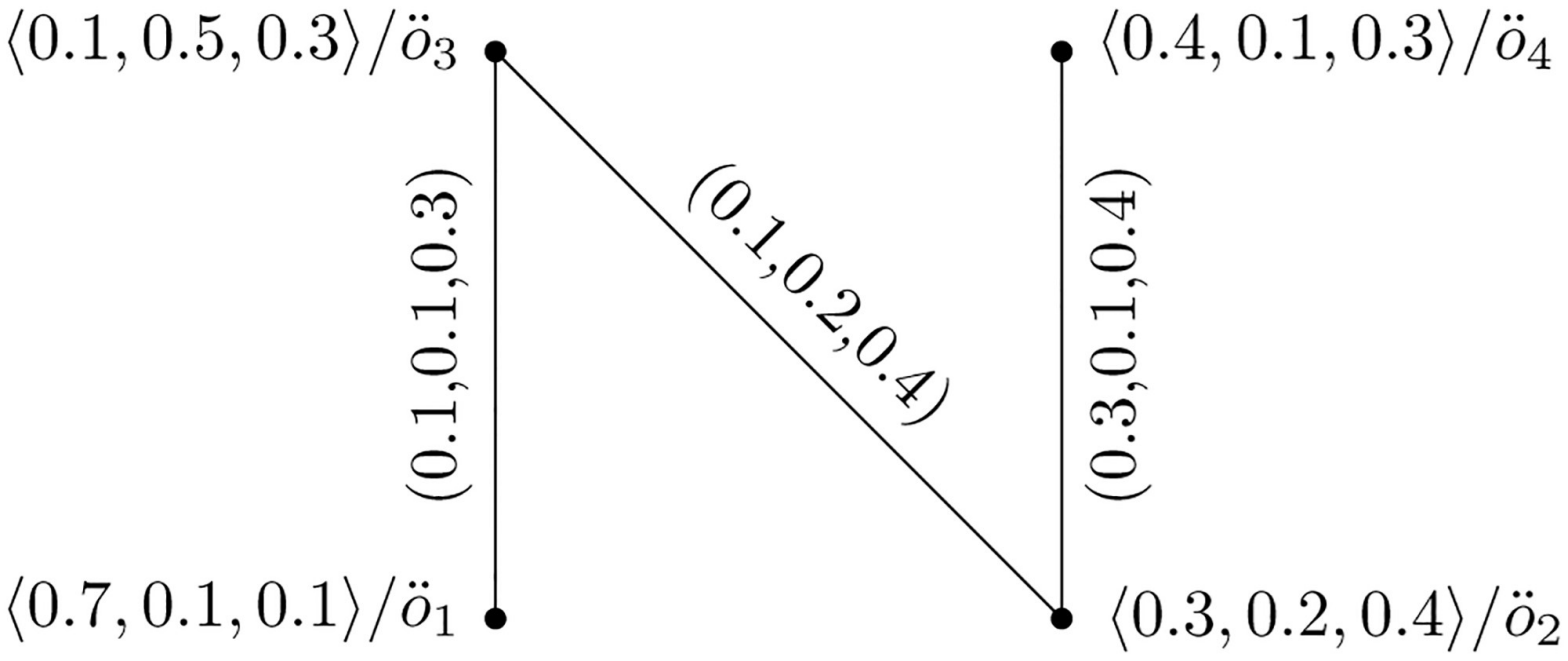
Picture fuzzy hypersoft graph $Y_{2}=\left(\ddot{H}\left({\ddot{t}}_{2}\right),\ddot{G}\left({\ddot{t}}_{2}\right)\right)$.

The PFHS graphs $Y_{1}=\left(\ddot{H}\left({\ddot{t}}_{1}\right),\ddot{G}\left({\ddot{t}}_{1}\right)\right)$, and $Y_{2}=\left(\ddot{H}\left({\ddot{t}}_{2}\right),\ddot{G}\left({\ddot{t}}_{2}\right)\right)$ are shown in Figs 1 and 2, respectively.

**Definition 13: Strong Picture Fuzzy Hypersoft Graph**

**Fig 3:**
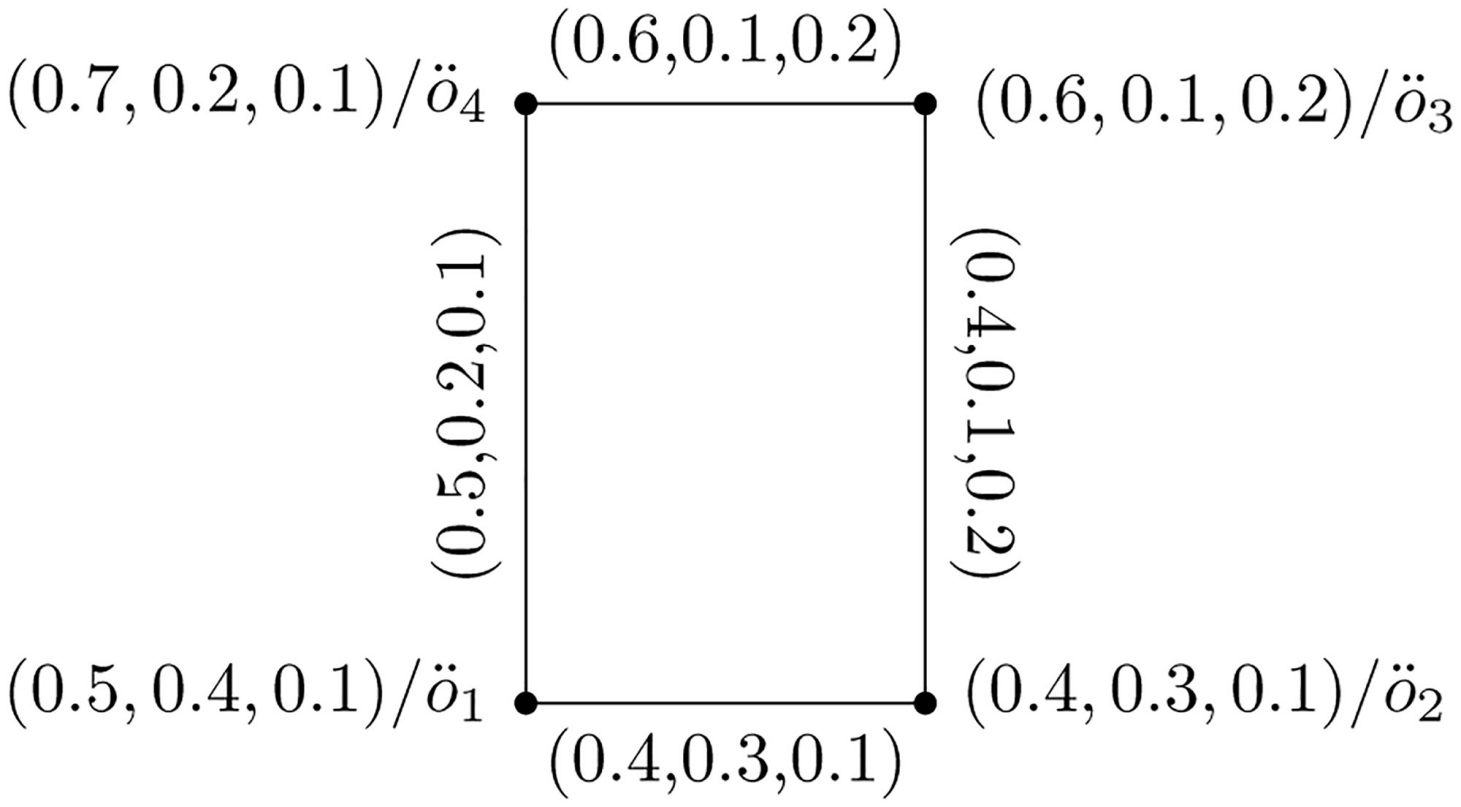
Strong picture fuzzy hypersoft graph.

A PFHSS graph Y=(U,W) is said to be strong if ${\overset{˘}{\mu }}_{W}\left(m,n\right)={\overset{˘}{\mu }}_{U}\left(m\right)\wedge {\overset{˘}{\mu }}_{U}\left(n\right),\;\;{\overset{˘}{\mu }}_{W}\left(m,n\right)={\overset{˘}{\mu }}_{U}\left(m\right)\wedge {\overset{˘}{\mu }}_{U}\left(n\right),\;{\overset{˘}{\mu }}_{W}\left(m,n\right)={\overset{˘}{\mu }}_{U}\left(m\right)\vee {\overset{˘}{\mu }}_{U}\left(n\right).$ Fig 3 represents the above presented structure with its edge set presented in Table 2.

**Table 2:** Edge set of PFHSG.

W	${\ddot{t}}_{1}$	${\ddot{t}}_{2}$	${\ddot{t}}_{3}$
${\ddot{o}}_{1}{\ddot{o}}_{2}$	〈0.3, 0.1, 0.4〉	〈0.0, 0.0, 0.0〉	〈0.0, 0.0, 0.0〉
${\ddot{o}}_{1}{\ddot{o}}_{3}$	〈0.1, 0.1, 0.3〉	〈0.1, 0.1, 0.3〉	〈0.1, 0.1, 0.3〉
${\ddot{o}}_{1}{\ddot{o}}_{4}$	〈0.4, 0.1, 0.3〉	〈0.0, 0.0, 0.0〉	〈0.4, 0.1, 0.3〉
${\ddot{o}}_{2}{\ddot{o}}_{3}$	〈0.0, 0.0, 0.0〉	〈0.1, 0.2, 0.4〉	〈0.0, 0.0, 0.0〉
${\ddot{o}}_{2}{\ddot{o}}_{4}$	〈0.0, 0.0, 0.0〉	〈0.3, 0.1, 0.4〉	〈0.0, 0.0, 0.0〉
${\ddot{o}}_{3}{\ddot{o}}_{4}$	〈0.0, 0.0, 0.0〉	〈0.0, 0.0, 0.0〉	〈0.1, 0.1, 0.3〉

**Definition 14: Complete Picture Fuzzy Hypersoft Graph**

**Fig 4:**
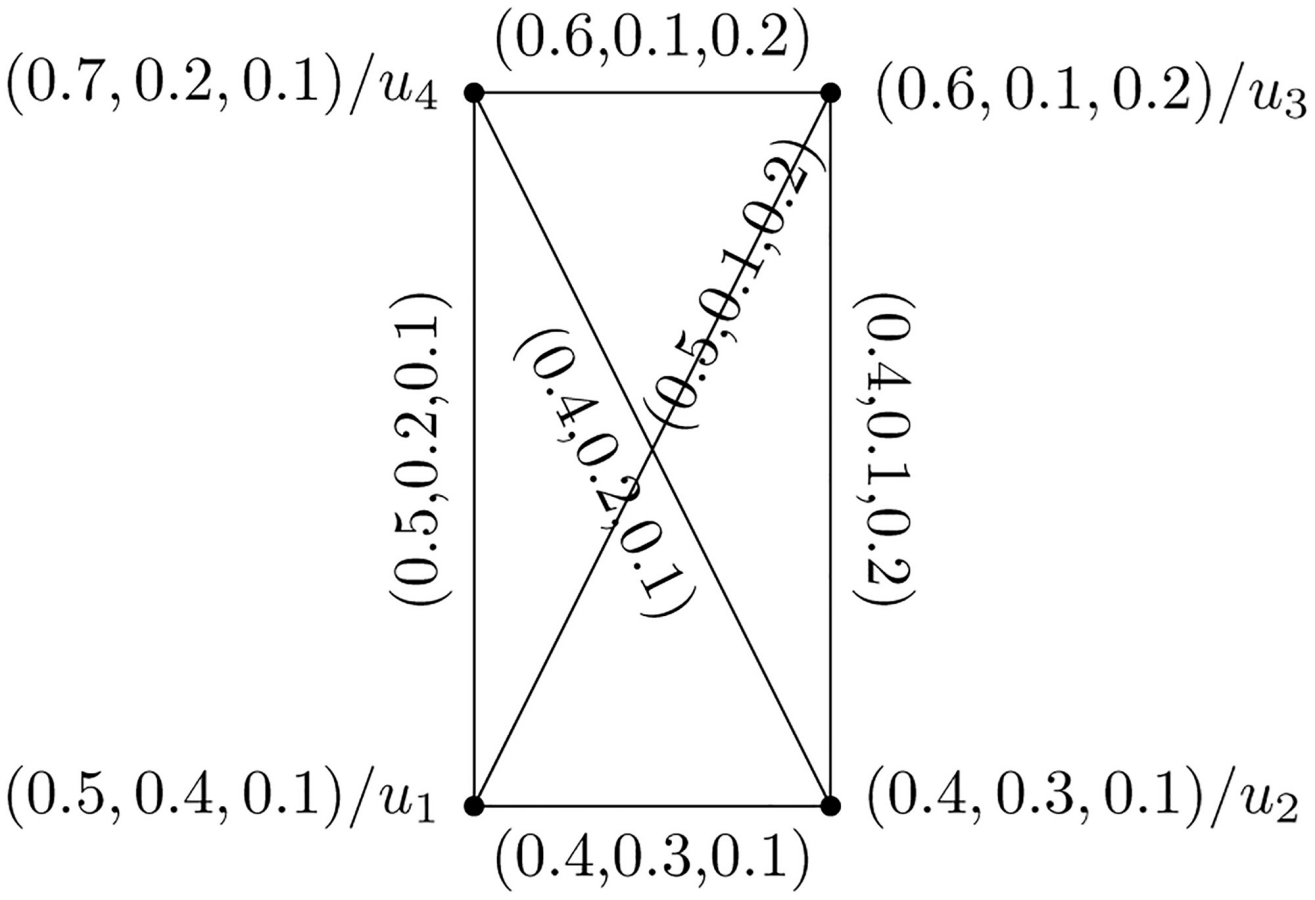
Complete picture fuzzy hypersoft graph.

A PFHSS graph Y=(U,W) is said to be complete if an edge lies between every two vertices of Y. ${\overset{˘}{\mu }}_{W}\left(m,n\right)={\overset{˘}{\mu }}_{U}\left(m\right)\wedge {\overset{˘}{\mu }}_{U}\left(n\right),\;\;{\overset{˘}{\mu }}_{W}\left(m,n\right)={\overset{˘}{\mu }}_{U}\left(m\right)\wedge {\overset{˘}{\mu }}_{U}\left(n\right),\;\;{\overset{˘}{\mu }}_{W}\left(m,n\right)={\overset{˘}{\mu }}_{U}\left(m\right)\vee {\overset{˘}{\mu }}_{U}\left(n\right).$ Fig 4 represents the above presented structure.

**Definition 15: Cartesian Product of Picture Fuzzy Hypersoft Graphs**

Suppose ${Y_{1}}^{⋆}=\left(V_{1},E_{1}\right)$ and ${Y_{1}}^{⋆}=\left(V_{2},E_{2}\right)$ be two graph. The Cartesian product of two PFHSG $Y_{1}=\left(U_{1},W_{1}\right)$ and $Y_{2}=\left(U_{2},W_{2}\right)$ is denoted $Y_{1}\times Y_{2}$ and defined by (U,W) where $U=({\overset{˘}{\mu }}_{U},{\overset{˘}{\eta }}_{U},{\overset{˘}{\nu }}_{U})$ and $W=({\overset{˘}{\mu }}_{W},{\overset{˘}{\eta }}_{W},{\overset{˘}{\nu }}_{W})$ are two PFHS on $V=V_{1}\times V_{2}$, and $E=\left\{\left(l,l_{2}\right),\left(l,m_{2}\right)|\;l\in V_{1},\;l_{2}m_{2}\in E_{2}\right\}\cup \left\{\left(l_{1},n\right),\left(m_{1},n\right)|n\in V_{2},\;l_{1}m_{1}\in E_{1}\right\}$ respectively which satisfies the following condition:

(i) For all $\left(l_{1},l_{2}\right)\in V_{1}\times V_{2}$
  (a) $\;\;{\overset{˘}{\mu }}_{U}\left(l_{1},l_{2}\right)={\overset{˘}{\mu }}_{U_{1}}\left(l_{1}\right)\wedge {\overset{˘}{\mu }}_{U_{1}}\left(l_{2}\right)$
  (b) $\;\;{\overset{˘}{\eta }}_{U}\left(l_{1},l_{2}\right)={\overset{˘}{\eta }}_{U_{1}}\left(l_{1}\right)\wedge {\overset{˘}{\eta }}_{U_{1}}\left(l_{2}\right)$
  (c) $\;\;{\overset{˘}{\nu }}_{U}\left(l_{1},l_{2}\right)={\overset{˘}{\nu }}_{U_{1}}\left(l_{1}\right)\vee {\overset{˘}{\nu }}_{U_{1}}\left(l_{2}\right)$
(ii) For all $l_{1}\in V_{1}$ and $\left(l_{2},m_{2}\right)\in E_{2}$
  (a) ${\overset{˘}{\mu }}_{W}\left(\left(l_{1},l_{2}\right)\left(l_{1},m_{2}\right)\right)={\overset{˘}{\mu }}_{U_{1}}\left(l_{1}\right)\wedge {\overset{˘}{\mu }}_{W_{2}}\left(l_{2}m_{2}\right)$
  (b) ${\overset{˘}{\eta }}_{W}\left(\left(l_{1},l_{2}\right)\left(l_{1},m_{2}\right)\right)={\overset{˘}{\eta }}_{U_{1}}\left(l_{1}\right)\wedge {\overset{˘}{\eta }}_{W_{2}}\left(l_{2}m_{2}\right)$
  (c) ${\overset{˘}{\nu }}_{W}\left(\left(l_{1},l_{2}\right)\left(l_{1},m_{2}\right)\right)={\overset{˘}{\nu }}_{U_{1}}\left(l_{1}\right)\vee {\overset{˘}{\nu }}_{W_{2}}\left(l_{2}m_{2}\right)$
(iii) For all $n\in V_{2}$ and $\left(l_{2},m_{2}\right)\in E_{1}$
  (a) ${\overset{˘}{\mu }}_{W}\left(\left(l_{1},l_{2}\right)\left(l_{1},m_{2}\right)\right)={\overset{˘}{\mu }}_{U_{2}}\left(n\right)\wedge {\overset{˘}{\mu }}_{W_{1}}\left(l_{1}m_{1}\right)$
  (b) ${\overset{˘}{\eta }}_{W}\left(\left(l_{1},l_{2}\right)\left(l_{1},m_{2}\right)\right)={\overset{˘}{\eta }}_{U_{2}}\left(n\right)\wedge {\overset{˘}{\eta }}_{W_{1}}\left(l_{1}m_{1}\right)$
  (c) ${\overset{˘}{\nu }}_{W}\left(\left(l_{1},l_{2}\right)\left(l_{1},m_{2}\right)\right)={\overset{˘}{\nu }}_{U_{2}}\left(n\right)\vee {\overset{˘}{\nu }}_{W_{1}}\left(l_{1}m_{1}\right)$

**Fig 5:**
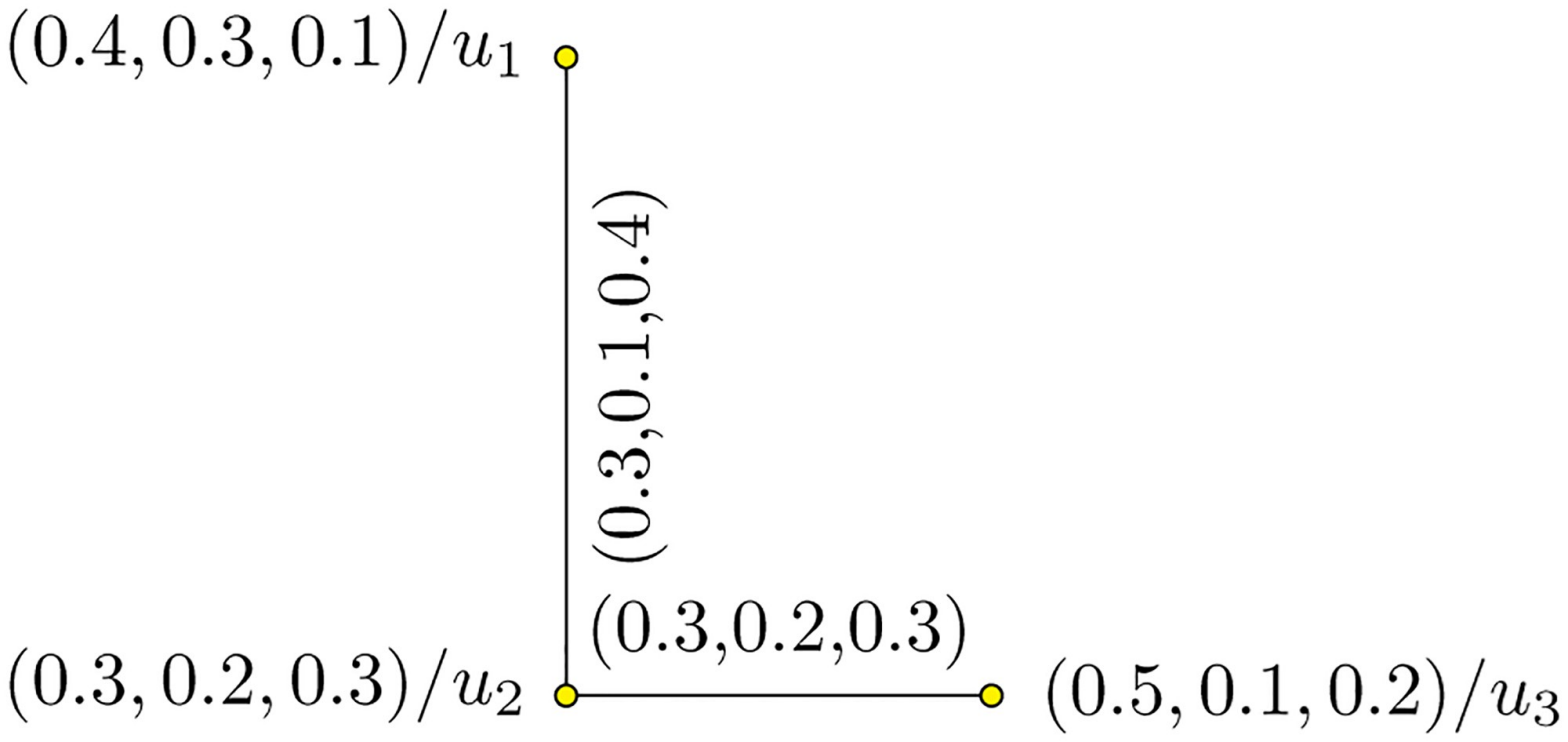
$Y_{1}=\left(U_{1},W_{1}\right)$ .

**Fig 6:**
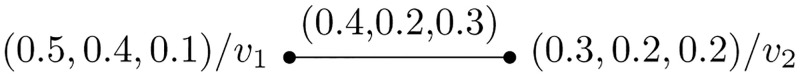
$Y_{2}=\left(U_{2},W_{2}\right)$ .

**Fig 7:**
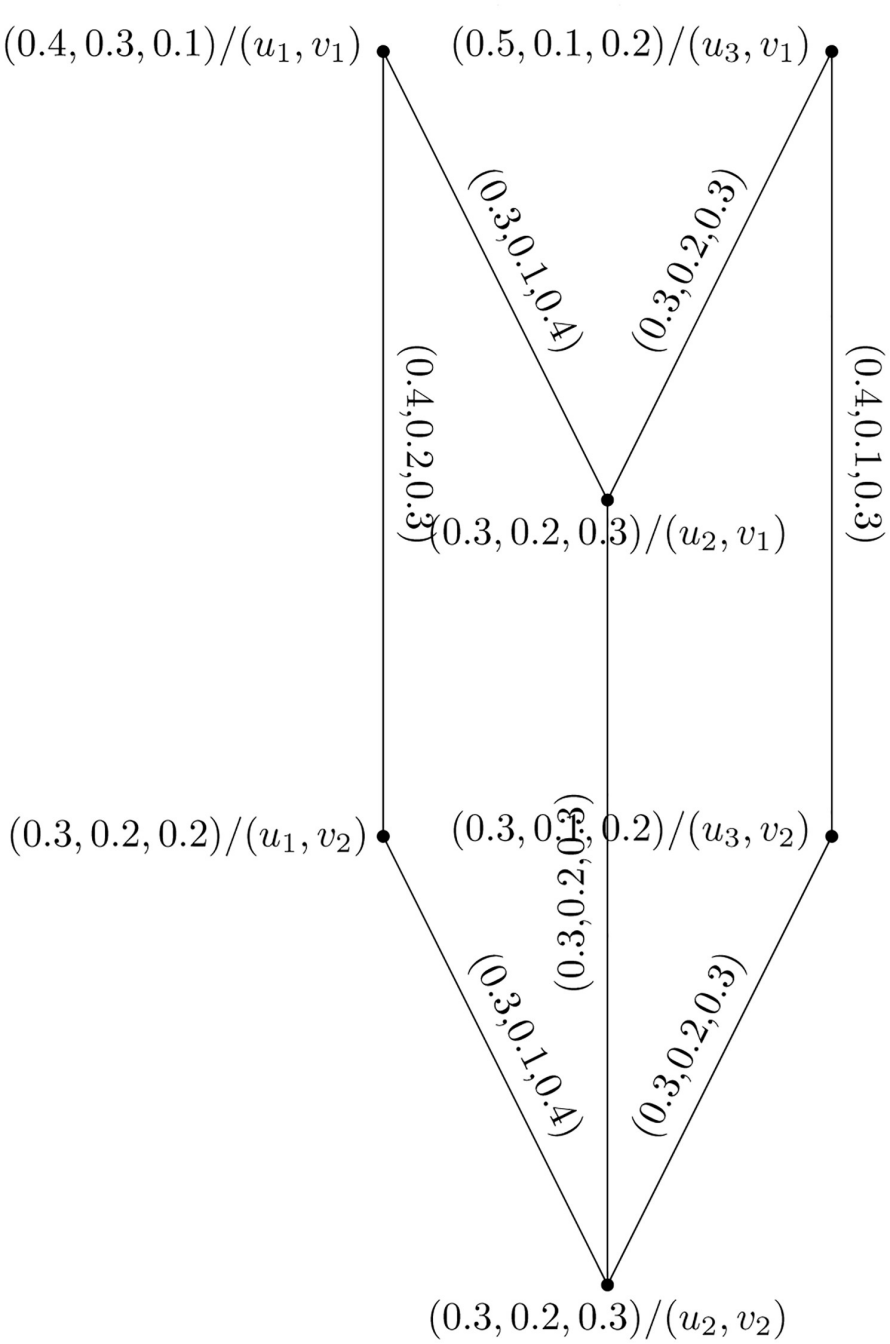
Cartesian product of two picture fuzzy hypersoft graphs.

Consider two PFHSG $Y_{1}=\left(U_{1},W_{1}\right)$ and $Y_{2}=\left(U_{2},W_{2}\right)$ are shown in Figs 5 and 6. The cartesian product of $Y_{1}=\left(U_{1},W_{1}\right)$ and $Y_{2}=\left(U_{2},W_{2}\right)$ is shown in Fig 7.

**Definition 16: Composition of Picture Fuzzy Hypersoft Graphs**

Suppose ${Y_{1}}^{⋆}=\left(V_{1},E_{1}\right)$ and ${Y_{1}}^{⋆}=\left(V_{2},E_{2}\right)$ be two graphs. The Composition of $Y_{1}=\left(W_{1},U_{1}\right)$ and $Y_{2}=\left(W_{2},U_{2}\right)$ is denoted $Y_{1}\left|Y_{2}\right|$ and defined by (W,U), where $W=({\overset{˘}{\mu }}_{W},{\overset{˘}{\eta }}_{W},{\overset{˘}{\nu }}_{W})$ and $U=({\overset{˘}{\mu }}_{U},{\overset{˘}{\eta }}_{U},{\overset{˘}{\nu }}_{U})$ are two PFS on $V=V_{1}\times V_{2}$, and $E=\left\{\left(l,l_{2}\right),\left(l,m_{2}\right)|\;l\in V_{1},\;l_{2}m_{2}\in E_{2}\right\}\cup \left\{\left(l_{1},n\right),\left(m_{1},n\right)|n\in V_{2},\;l_{1}m_{1}\in E_{1}\right\}\cup \left\{\left(l_{1},l_{2}\right),\left(m_{1},m_{2}\right)|\;l_{2}m_{2}\in V_{2}\;l_{2}\neq m_{2},\;l_{1}m_{1}\in E_{1}\right\}$ respectively which satisfy the following conditions:

(i) For all $\left(l_{1},l_{2}\right)\in V_{1}\times V_{2}$
  (a) $\;\;{\overset{˘}{\mu }}_{W}\left(l_{1},l_{2}\right)={\overset{˘}{\mu }}_{W_{1}}\left(l_{1}\right)\wedge {\overset{˘}{\mu }}_{W_{1}}\left(l_{2}\right)$
  (b) $\;\;{\overset{˘}{\eta }}_{W}\left(l_{1},l_{2}\right)={\overset{˘}{\eta }}_{W_{1}}\left(l_{1}\right)\wedge {\overset{˘}{\eta }}_{W_{1}}\left(l_{2}\right)$
  (c) $\;\;{\overset{˘}{\nu }}_{W}\left(l_{1},l_{2}\right)={\overset{˘}{\nu }}_{W_{1}}\left(l_{1}\right)\vee {\overset{˘}{\nu }}_{W_{1}}\left(l_{2}\right)$
(ii) For all $l\in V_{1}$ and $\left(l_{2},m_{2}\right)\in E_{2}$
  (a) ${\overset{˘}{\mu }}_{U}\left(\left(l_{1},l_{2}\right)\left(l_{1},m_{2}\right)\right)={\overset{˘}{\mu }}_{W_{1}}\left(l_{1}\right)\wedge {\overset{˘}{\mu }}_{U_{2}}\left(l_{2}m_{2}\right)$
  (b) ${\overset{˘}{\eta }}_{U}\left(\left(l_{1},l_{2}\right)\left(l_{1},m_{2}\right)\right)={\overset{˘}{\eta }}_{W_{1}}\left(l_{1}\right)\wedge {\overset{˘}{\eta }}_{U_{2}}\left(l_{2}m_{2}\right)$
  (c) ${\overset{˘}{\nu }}_{U}\left(\left(l_{1},l_{2}\right)\left(l_{1},m_{2}\right)\right)={\overset{˘}{\nu }}_{W_{1}}\left(l_{1}\right)\vee {\overset{˘}{\nu }}_{U_{2}}\left(l_{2}m_{2}\right)$
(iii) For all $n\in V_{2}$ and $\left(l_{2},m_{2}\right)\in E_{1}$
  (a) ${\overset{˘}{\mu }}_{U}\left(\left(l_{1},l_{2}\right)\left(l_{1},m_{2}\right)\right)={\overset{˘}{\mu }}_{W_{2}}\left(n\right)\wedge {\overset{˘}{\mu }}_{U_{1}}\left(l_{1}m_{1}\right)$
  (b) ${\overset{˘}{\eta }}_{U}\left(\left(l_{1},l_{2}\right)\left(l_{1},m_{2}\right)\right)={\overset{˘}{\eta }}_{W_{2}}\left(n\right)\wedge {\overset{˘}{\eta }}_{U_{1}}\left(l_{1}m_{1}\right)$
  (c) ${\overset{˘}{\nu }}_{U}\left(\left(l_{1},l_{2}\right)\left(l_{1},m_{2}\right)\right)={\overset{˘}{\nu }}_{W_{2}}\left(n\right)\vee {\overset{˘}{\nu }}_{U_{1}}\left(l_{1}m_{1}\right)$
(iv) For all $l_{2}m_{2}\in V_{2}\;\;l_{2}\neq m_{2}$ and $\left(l_{1},m_{1}\right)\in E_{1}$
  (a) ${\overset{˘}{\mu }}_{U}\left(\left(l_{1},l_{2}\right)\left(m_{1},m_{2}\right)\right)={\overset{˘}{\mu }}_{W_{2}}\left(l_{2}\right)\wedge {\overset{˘}{\mu }}_{W_{2}}\left(m_{2}\right)\wedge {\overset{˘}{\mu }}_{U_{1}}\left(l_{1}m_{1}\right)$
  (b) ${\overset{˘}{\eta }}_{U}\left(\left(l_{1},l_{2}\right)\left(m_{1},m_{2}\right)\right)={\overset{˘}{\eta }}_{W_{2}}\left(l_{2}\right)\wedge {\overset{˘}{\eta }}_{W_{2}}\left(m_{2}\right)\wedge {\overset{˘}{\eta }}_{U_{1}}\left(l_{1}m_{1}\right)$
  (c) ${\overset{˘}{\nu }}_{U}\left(\left(l_{1},l_{2}\right)\left(m_{1},m_{2}\right)\right)={\overset{˘}{\nu }}_{W_{2}}\left(l_{2}\right)\wedge {\overset{˘}{\nu }}_{W_{2}}\left(m_{2}\right)\wedge {\overset{˘}{\nu }}_{U_{1}}\left(l_{1}m_{1}\right)$

**Fig 8:**
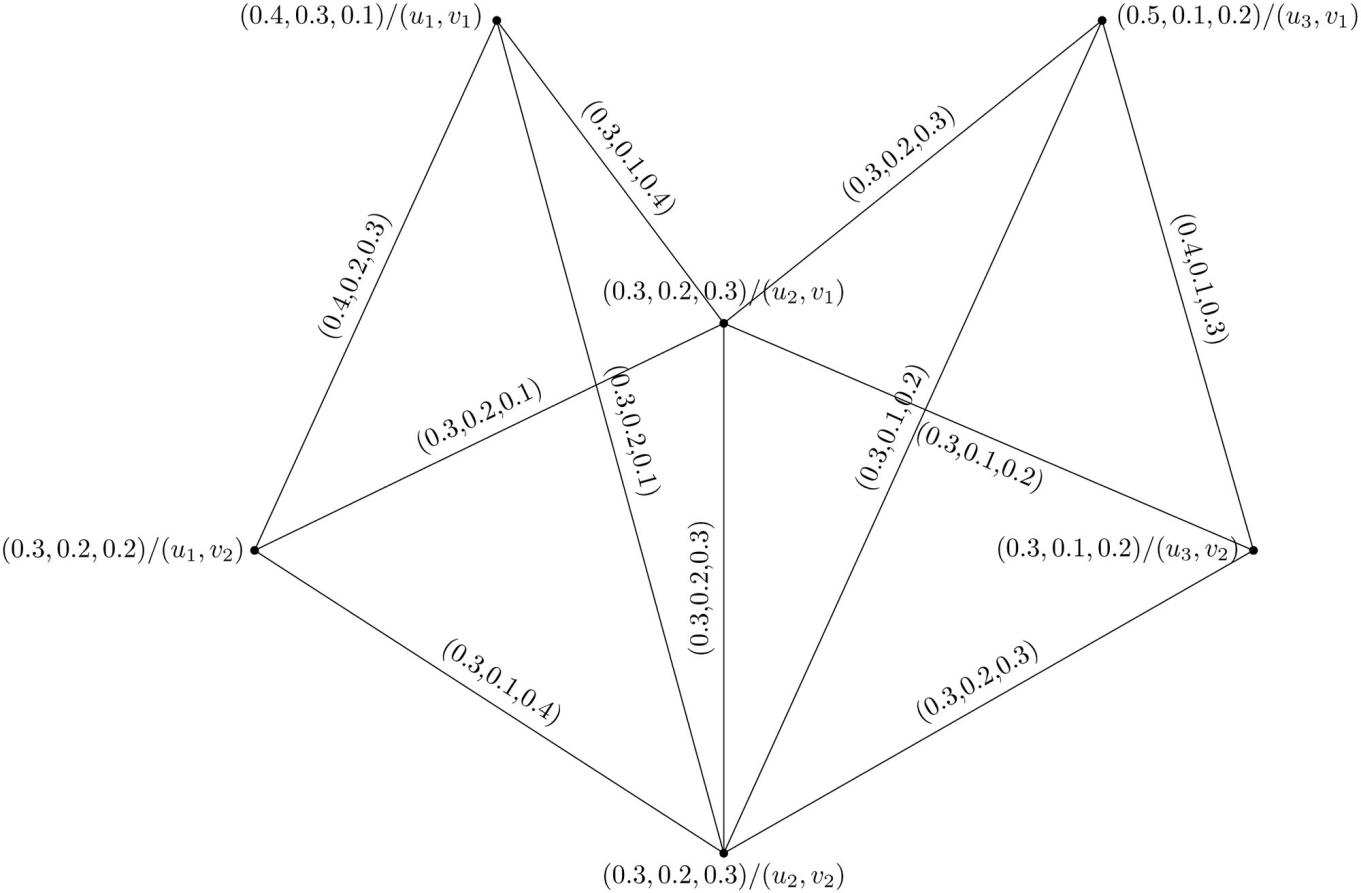
Composition of picture fuzzy hypersoft graphs.

The composition of $Y_{1}=\left(U_{1},W_{1}\right)$ and $Y_{2}=\left(U_{2},W_{2}\right)$ is shown in Fig 8.

**Definition 17: Union of Picture Fuzzy Hypersoft Graphs**

Suppose ${Y_{1}}^{⋆}=\left(V_{1},E_{1}\right)$ and ${Y_{1}}^{⋆}=\left(V_{2},E_{2}\right)$ be two graph. The union of two PFGs $Y_{1}=\left(W_{1},U_{1}\right)$ and $Y_{2}=\left(W_{2},U_{2}\right)$ is denoted $Y_{1}\cup Y_{2}$ and defined by (W,U) where $W=({\overset{˘}{\mu }}_{W},{\overset{˘}{\eta }}_{W},{\overset{˘}{\nu }}_{W})$ on $V=V_{1}\cup V_{2}$ and $U=({\overset{˘}{\mu }}_{U},{\overset{˘}{\eta }}_{U},{\overset{˘}{\nu }}_{U})$ on $E=E_{1}\cup E_{2}$.

(i) (a) $\;\;{\overset{˘}{\mu }}_{W}\left(l\right)={\overset{˘}{\mu }}_{W_{1}}\left(l\right)\;\;l\in V_{1}-V_{2}$
  (b) $\;\;{\overset{˘}{\mu }}_{W}\left(l\right)={\overset{˘}{\mu }}_{W_{2}}\left(l\right)\;\;l\in V_{2}-V_{1}$
  (c) $\;\;{\overset{˘}{\mu }}_{W}\left(l\right)={\overset{˘}{\mu }}_{W_{1}}\left(l\right)\wedge {\overset{˘}{\mu }}_{W_{2}}\left(l\right)\;\;l\in V_{1}\cap V_{2}$
(ii) (a) $\;\;{\overset{˘}{\eta }}_{W}\left(l\right)={\overset{˘}{\eta }}_{W_{1}}\left(l\right)\;\;l\in V_{1}-V_{2}$
  (b) $\;\;{\overset{˘}{\eta }}_{W}\left(l\right)={\overset{˘}{\eta }}_{W_{2}}\left(l\right)\;\;l\in V_{2}-V_{1}$
  (c) $\;\;{\overset{˘}{\eta }}_{W}\left(l\right)={\overset{˘}{\eta }}_{W_{1}}\left(l\right)\wedge {\overset{˘}{\eta }}_{W_{2}}\left(l\right)\;\;l\in V_{1}\cap V_{2}$
(iii) (a) $\;\;{\overset{˘}{\nu }}_{W}\left(l\right)={\overset{˘}{\nu }}_{W_{1}}\left(l\right)\;\;l\in V_{1}-V_{2}$
  (b) $\;\;{\overset{˘}{\nu }}_{W}\left(l\right)={\overset{˘}{\nu }}_{W_{2}}\left(l\right)\;\;l\in V_{2}-V_{1}$
  (c) $\;\;{\overset{˘}{\nu }}_{W}\left(l\right)={\overset{˘}{\nu }}_{W_{1}}\left(l\right)\wedge {\overset{˘}{\nu }}_{W_{2}}\left(l\right)\;\;l\in V_{1}\cap V_{2}$
(iv) (a) $\;\;{\overset{˘}{\mu }}_{W}\left(lm\right)={\overset{˘}{\mu }}_{U_{1}}\left(lm\right)\;\;lm\in E_{1}-E_{2}$
  (b) $\;\;{\overset{˘}{\mu }}_{U}\left(lm\right)={\overset{˘}{\mu }}_{U_{2}}\left(lm\right)\;\;l\in E_{2}-E_{1}$
  (c) $\;\;{\overset{˘}{\mu }}_{U}\left(lm\right)={\overset{˘}{\mu }}_{U_{1}}\left(lm\right)\wedge {\overset{˘}{\mu }}_{U_{2}}\left(lm\right)\;\;l\in E_{1}\cap E_{2}$
(v) (a) $\;\;{\overset{˘}{\eta }}_{W}\left(lm\right)={\overset{˘}{\eta }}_{U_{1}}\left(lm\right)\;\;lm\in E_{1}-E_{2}$
  (b) $\;\;{\overset{˘}{\eta }}_{U}\left(lm\right)={\overset{˘}{\eta }}_{U_{2}}\left(lm\right)\;\;l\in E_{2}-E_{1}$
  (c) $\;\;{\overset{˘}{\eta }}_{U}\left(lm\right)={\overset{˘}{\eta }}_{U_{1}}\left(lm\right)\wedge {\overset{˘}{\eta }}_{U_{2}}\left(lm\right)\;\;l\in E_{1}\cap E_{2}$
(vi) (a) $\;\;{\overset{˘}{\nu }}_{W}\left(lm\right)={\overset{˘}{\nu }}_{U_{1}}\left(lm\right)\;\;lm\in E_{1}-E_{2}$
  (b) $\;\;{\overset{˘}{\nu }}_{U}\left(lm\right)={\overset{˘}{\nu }}_{U_{2}}\left(lm\right)\;\;l\in E_{2}-E_{1}$
  (c) $\;\;{\overset{˘}{\nu }}_{U}\left(lm\right)={\overset{˘}{\nu }}_{U_{1}}\left(lm\right)\wedge {\overset{˘}{\nu }}_{U_{2}}\left(lm\right)\;\;l\in E_{1}\cap E_{2}$

**Fig 9:**
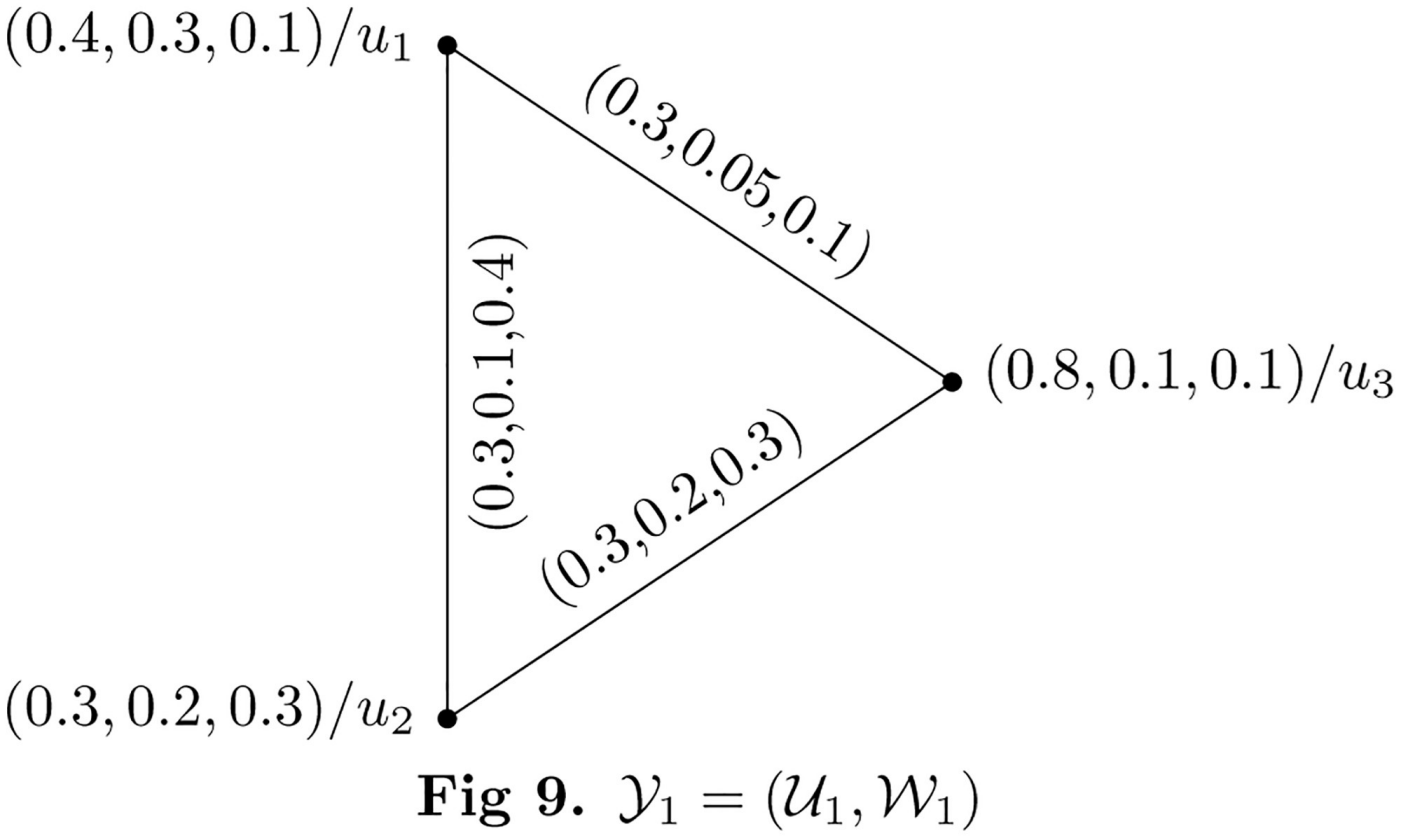
$Y_{1}=\left(U_{1},W_{1}\right)$ .

**Fig 10:**
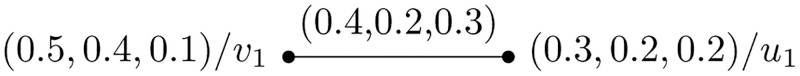
$Y_{2}=\left(U_{2},W_{2}\right)$ .

**Fig 11:**
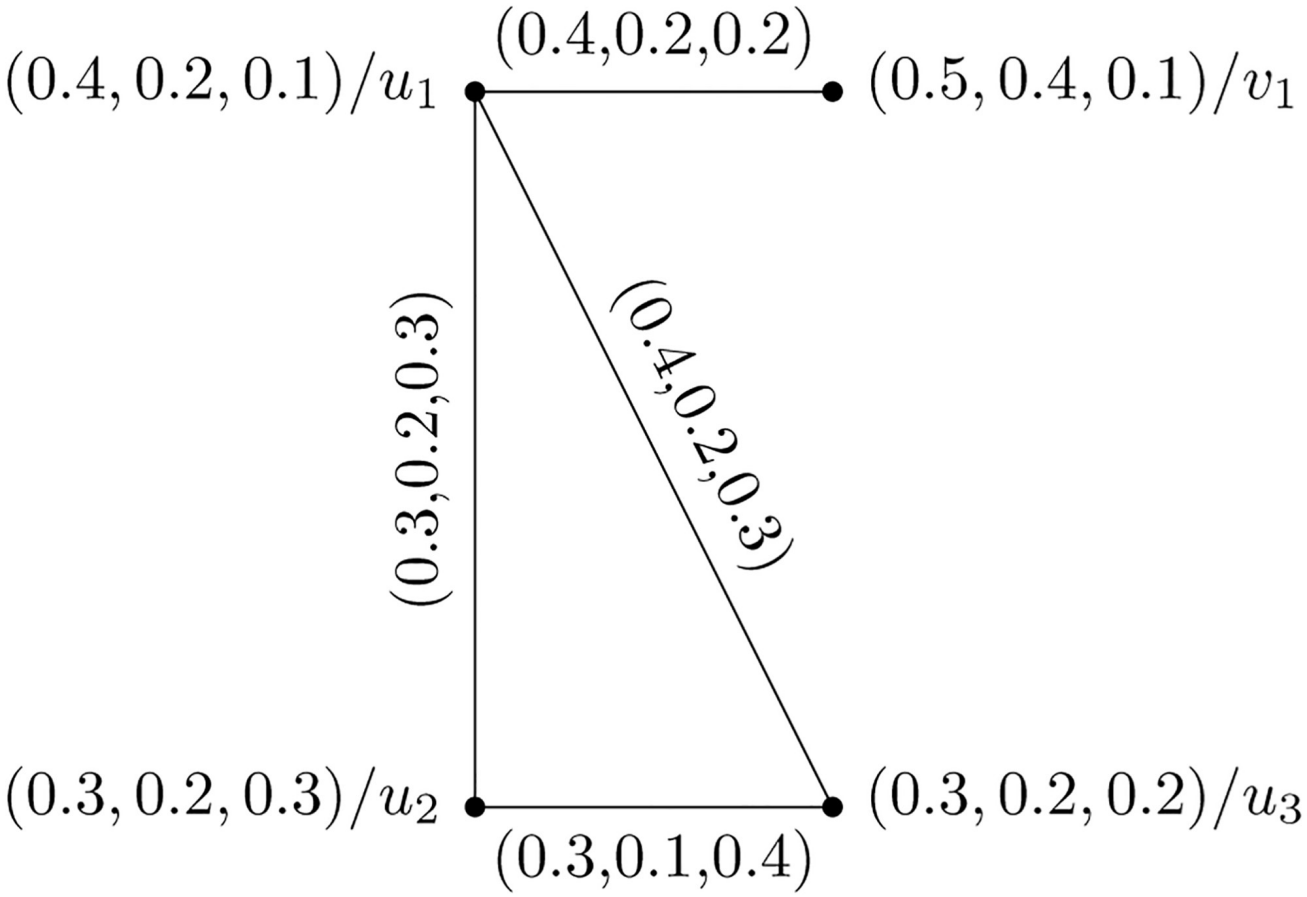
Union of PFHSG.

Consider two PFHSG $Y_{1}=\left(U_{1},W_{1}\right)$ and $Y_{2}=\left(U_{2},W_{2}\right)$ are shown in Figs 9 and 10. The union of $Y_{1}=\left(U_{1},W_{1}\right)$ and $Y_{2}=\left(U_{2},W_{2}\right)$ is shown in Fig 11.

**Definition 18: Joint of two Picture Fuzzy Hypersoft Graphs**

Suppose ${Y_{1}}^{⋆}=\left(V_{1},E_{1}\right)$ and ${Y_{1}}^{⋆}=\left(V_{2},E_{2}\right)$ be two graphs. The joint of two PFHSGs $Y_{1}=\left(W_{1},U_{1}\right)$ and $Y_{2}=\left(W_{2},U_{2}\right)$ is denoted $Y_{1}+Y_{2}$ and defined by (W,U) where $W=({\overset{˘}{\mu }}_{W},{\overset{˘}{\eta }}_{W},{\overset{˘}{\nu }}_{W})$ on $V=V_{1}\cup V_{2}$ and $U=({\overset{˘}{\mu }}_{U},{\overset{˘}{\eta }}_{U},{\overset{˘}{\nu }}_{U})$ on $E=E_{1}\cup E_{2}\cup E_{3}$.

(i) (a) $\;\;{\overset{˘}{\mu }}_{W}\left(l\right)={\overset{˘}{\mu }}_{W_{1}}\left(l\right)\;\;l\in V_{1}-V_{2}$
  (b) $\;\;{\overset{˘}{\mu }}_{W}\left(l\right)={\overset{˘}{\mu }}_{W_{2}}\left(l\right)\;\;l\in V_{2}-V_{1}$
  (c) $\;\;{\overset{˘}{\mu }}_{W}\left(l\right)={\overset{˘}{\mu }}_{W_{1}}\left(l\right)\wedge {\overset{˘}{\mu }}_{W_{2}}\left(l\right)\;\;l\in V_{1}\cap V_{2}$
(ii) (a) $\;\;{\overset{˘}{\eta }}_{W}\left(l\right)={\overset{˘}{\eta }}_{W_{1}}\left(l\right)\;\;l\in V_{1}-V_{2}$
  (b) $\;\;{\overset{˘}{\eta }}_{W}\left(l\right)={\overset{˘}{\eta }}_{W_{2}}\left(l\right)\;\;l\in V_{2}-V_{1}$
  (c) $\;\;{\overset{˘}{\eta }}_{W}\left(l\right)={\overset{˘}{\eta }}_{W_{1}}\left(l\right)\wedge {\overset{˘}{\eta }}_{W_{2}}\left(l\right)\;\;l\in V_{1}\cap V_{2}$
(iii) (a) $\;\;{\overset{˘}{\nu }}_{W}\left(l\right)={\overset{˘}{\nu }}_{W_{1}}\left(l\right)\;\;l\in V_{1}-V_{2}$
  (b) $\;\;{\overset{˘}{\nu }}_{W}\left(l\right)={\overset{˘}{\nu }}_{W_{2}}\left(l\right)\;\;l\in V_{2}-V_{1}$
  (c) $\;\;{\overset{˘}{\nu }}_{W}\left(l\right)={\overset{˘}{\nu }}_{W_{1}}\left(l\right)\wedge {\overset{˘}{\nu }}_{W_{2}}\left(l\right)\;\;l\in V_{1}\cap V_{2}$
(iv) (a) $\;\;{\overset{˘}{\mu }}_{W}\left(lm\right)={\overset{˘}{\mu }}_{U_{1}}\left(lm\right)\;\;lm\in E_{1}-E_{2}$
  (b) $\;\;{\overset{˘}{\mu }}_{U}\left(lm\right)={\overset{˘}{\mu }}_{U_{2}}\left(lm\right)\;\;l\in E_{2}-E_{1}$
  (c) $\;\;{\overset{˘}{\mu }}_{U}\left(lm\right)={\overset{˘}{\mu }}_{U_{1}}\left(lm\right)\wedge {\overset{˘}{\mu }}_{U_{2}}\left(lm\right)\;\;l\in E_{1}\cap E_{2}$
(v) (a) $\;\;{\overset{˘}{\eta }}_{W}\left(lm\right)={\overset{˘}{\eta }}_{U_{1}}\left(lm\right)\;\;lm\in E_{1}-E_{2}$
  (b) $\;\;{\overset{˘}{\eta }}_{U}\left(lm\right)={\overset{˘}{\eta }}_{U_{2}}\left(lm\right)\;\;l\in E_{2}-E_{1}$
  (c) $\;\;{\overset{˘}{\eta }}_{U}\left(lm\right)={\overset{˘}{\eta }}_{U_{1}}\left(lm\right)\wedge {\overset{˘}{\eta }}_{U_{2}}\left(lm\right)\;\;l\in E_{1}\cap E_{2}$
(vi) (a) $\;\;{\overset{˘}{\nu }}_{W}\left(lm\right)={\overset{˘}{\nu }}_{U_{1}}\left(lm\right)\;\;lm\in E_{1}-E_{2}$
  (b) $\;\;{\overset{˘}{\nu }}_{U}\left(lm\right)={\overset{˘}{\nu }}_{U_{2}}\left(lm\right)\;\;l\in E_{2}-E_{1}$
  (c) $\;\;{\overset{˘}{\nu }}_{U}\left(lm\right)={\overset{˘}{\nu }}_{U_{1}}\left(lm\right)\wedge {\overset{˘}{\nu }}_{U_{2}}\left(lm\right)\;\;l\in E_{1}\cap E_{2}$
(vii) (a) $\;\;{\overset{˘}{\mu }}_{W}\left(lm\right)={\overset{˘}{\mu }}_{U_{1}}\left(lm\right)\vee {\overset{˘}{\mu }}_{U_{2}}\left(lm\right)\;\;lm\in E_{3}$
  (b) $\;\;{\overset{˘}{\eta }}_{W}\left(lm\right)={\overset{˘}{\eta }}_{U_{1}}\left(lm\right)\vee {\overset{˘}{\eta }}_{U_{2}}\left(lm\right)\;\;lm\in E_{3}$
  (c) $\;\;{\overset{˘}{\nu }}_{W}\left(lm\right)={\overset{˘}{\nu }}_{U_{1}}\left(lm\right)\wedge {\overset{˘}{\nu }}_{U_{2}}\left(lm\right)\;\;lm\in E_{3}$

**Fig 12:**
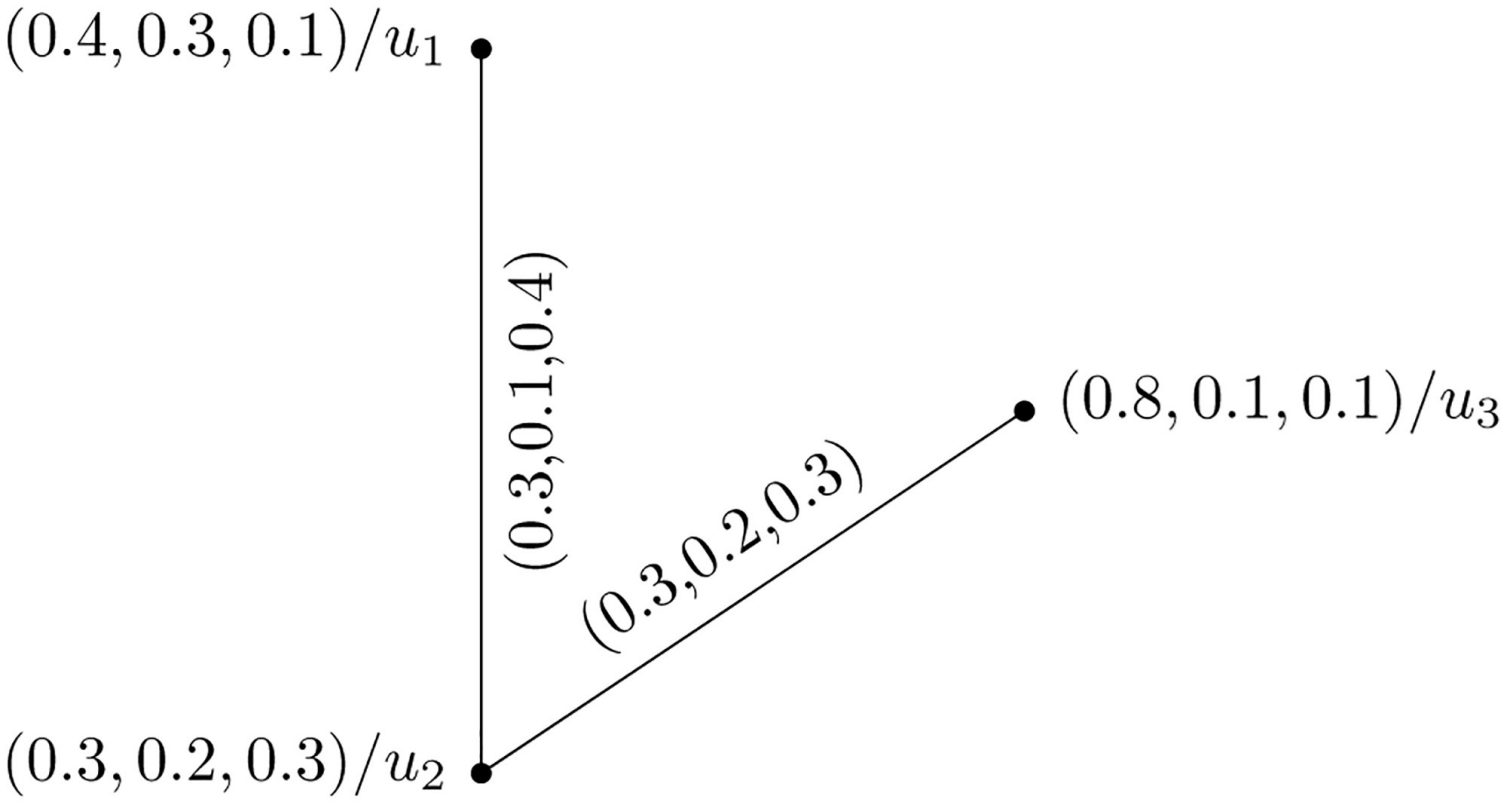
$Y_{1}=\left(U_{1},W_{1}\right)$ .

**Fig 13:**
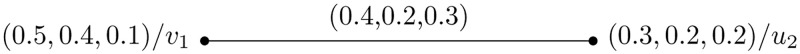
$Y_{2}=\left(U_{2},W_{2}\right)$ .

**Fig 14:**
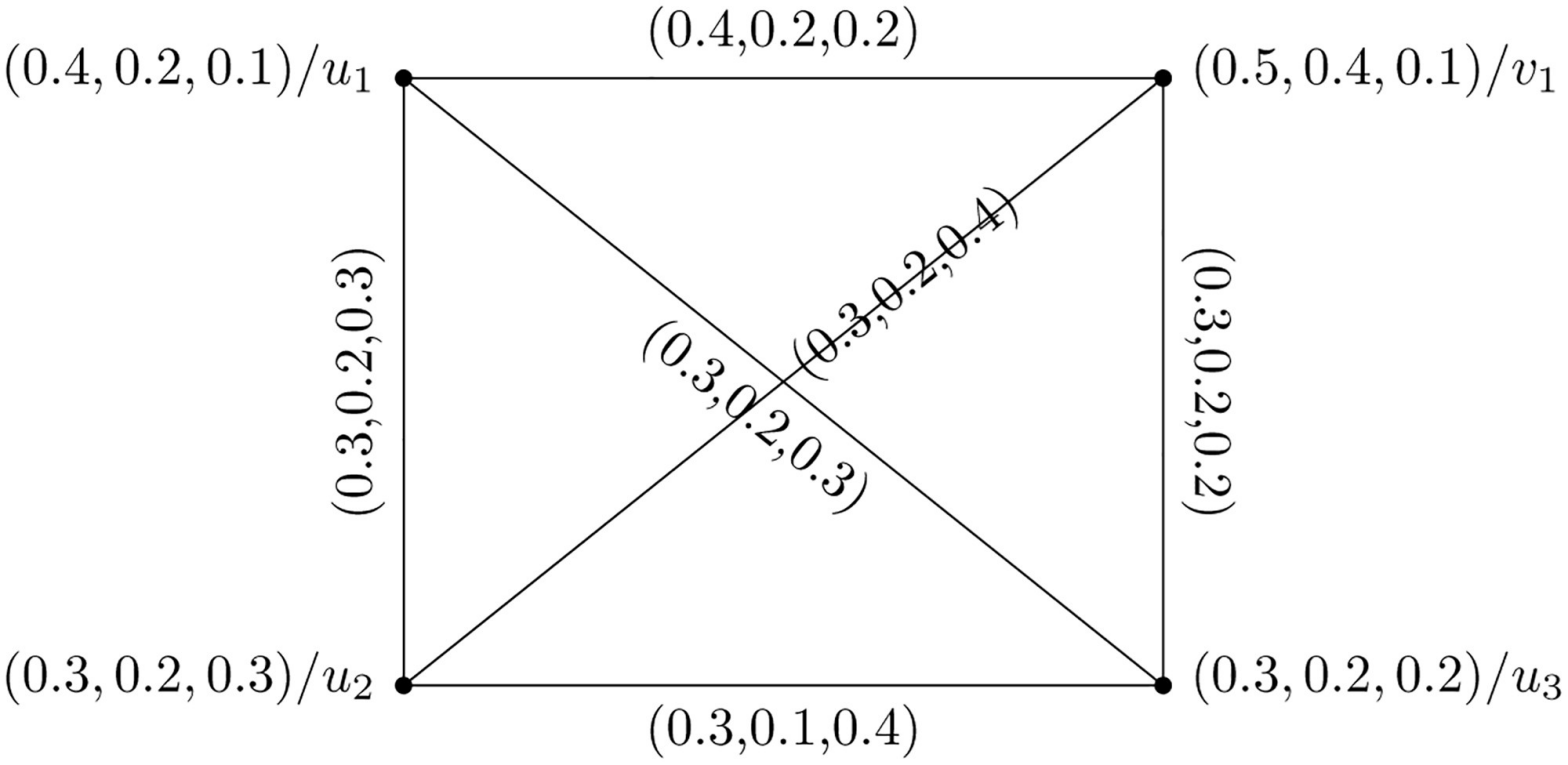
Join of PFHSG.

Consider two PFHSG $Y_{1}=\left(U_{1},W_{1}\right)$ and $Y_{2}=\left(U_{2},W_{2}\right)$ are shown in Figs 12 and 13. The joint of $Y_{1}=\left(U_{1},W_{1}\right)$ and $Y_{2}=\left(U_{2},W_{2}\right)$ is shown in Fig 14.

**Definition 19: Joint of Picture Fuzzy Hypersoft Graph**

Suppose ${Y_{1}}^{⋆}=\left(V_{1},E_{1}\right)$ and ${Y_{1}}^{⋆}=\left(V_{2},E_{2}\right)$ be two graph. The direct product of $Y_{1}=\left(W_{1},U_{1}\right)$ and $Y_{2}=\left(W_{2},U_{2}\right)$ is denoted $Y_{1}⊗Y_{2}$ and defined by (W,U) where $W=({\overset{˘}{\mu }}_{W},{\overset{˘}{\eta }}_{W},{\overset{˘}{\nu }}_{W})$ and $U=({\overset{˘}{\mu }}_{U},{\overset{˘}{\eta }}_{U},{\overset{˘}{\nu }}_{U})$ are two PFS on $V=V_{1}\times V_{2}$, and $E=\{\left(l_{1},m_{1}\right),\left(l_{2},m_{2}\right)|\;l_{1}m_{1}\in E_{1},\;l_{2}m_{2}\in E_{2}\}$ which satisfies the following condition

(i) For all $\left(l_{1},l_{2}\right)\in V_{1}\times V_{2}$
  (a) $\;\;{\overset{˘}{\mu }}_{W}\left(l_{1},l_{2}\right)={\overset{˘}{\mu }}_{W_{1}}\left(l_{1}\right)\wedge {\overset{˘}{\mu }}_{W_{1}}\left(l_{2}\right)$
  (b) $\;\;{\overset{˘}{\eta }}_{W}\left(l_{1},l_{2}\right)={\overset{˘}{\eta }}_{W_{1}}\left(l_{1}\right)\wedge {\overset{˘}{\eta }}_{W_{1}}\left(l_{2}\right)$
  (c) $\;\;{\overset{˘}{\nu }}_{W}\left(l_{1},l_{2}\right)={\overset{˘}{\nu }}_{W_{1}}\left(l_{1}\right)\vee {\overset{˘}{\nu }}_{W_{1}}\left(l_{2}\right)$
(ii) For all $\left(l_{1}m_{1}\right)\in E_{1}$ and $\left(l_{2}m_{2}\right)\in E_{2}$
  (a) ${\overset{˘}{\mu }}_{U}\left(\left(l_{1},l_{2}\right)\left(m_{1},m_{2}\right)\right)={\overset{˘}{\mu }}_{U_{1}}\left(l_{1}m_{1}\right)\wedge {\overset{˘}{\mu }}_{U_{2}}\left(l_{2}m_{2}\right)$
  (b) ${\overset{˘}{\eta }}_{U}\left(\left(l_{1},l_{2}\right)\left(m_{1},m_{2}\right)\right)={\overset{˘}{\eta }}_{U_{1}}\left(l_{1}m_{1}\right)\wedge {\overset{˘}{\eta }}_{U_{2}}\left(l_{2}m_{2}\right)$
  (c) ${\overset{˘}{\nu }}_{U}\left(\left(l_{1},l_{2}\right)\left(m_{1},m_{2}\right)\right)={\overset{˘}{\nu }}_{U_{1}}\left(l_{1}m_{1}\right)\vee {\overset{˘}{\nu }}_{U_{2}}\left(l_{2}m_{2}\right)$

**Fig 15:**
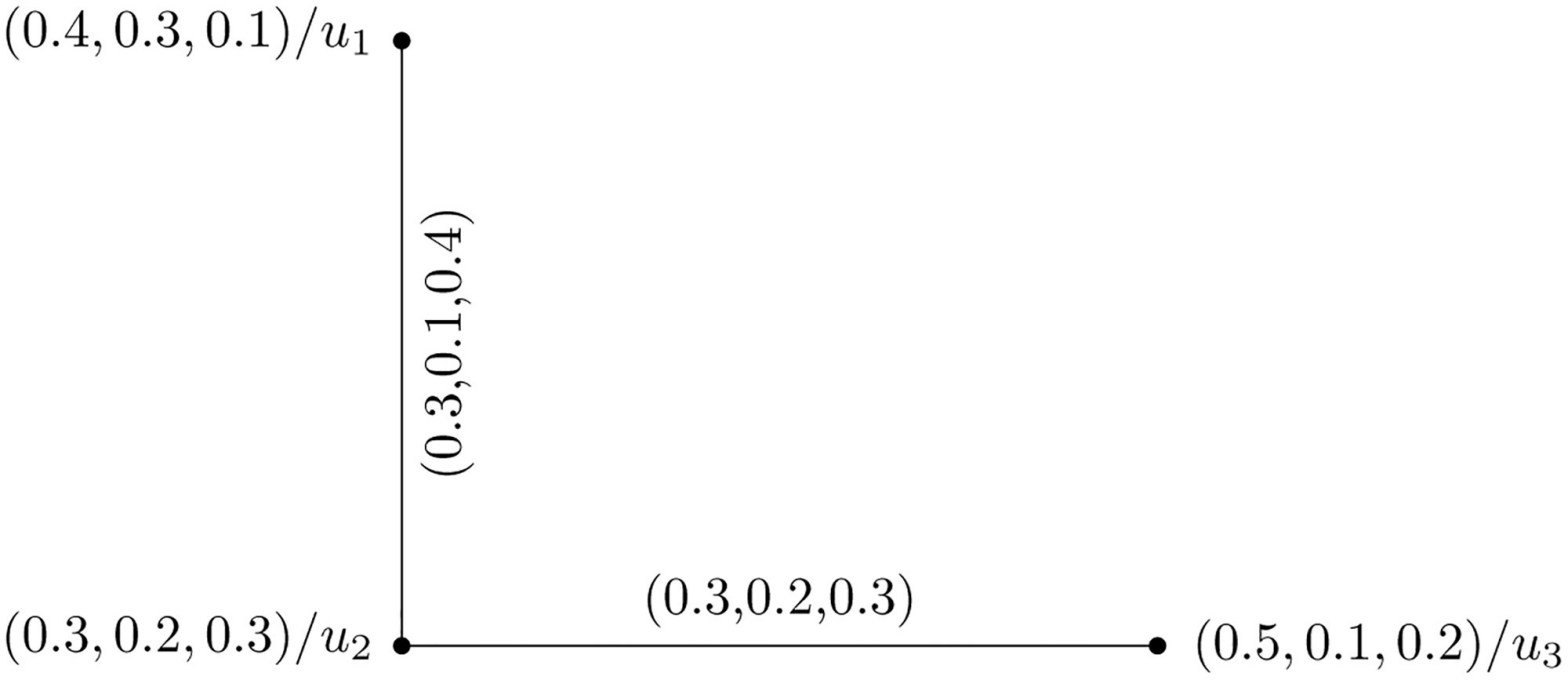
$Y_{1}=\left(U_{1},W_{1}\right)$ .

**Fig 16:**
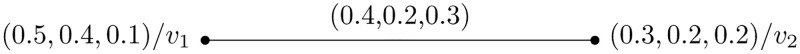
$Y_{2}=\left(U_{2},W_{2}\right)$ .

**Fig 17:**
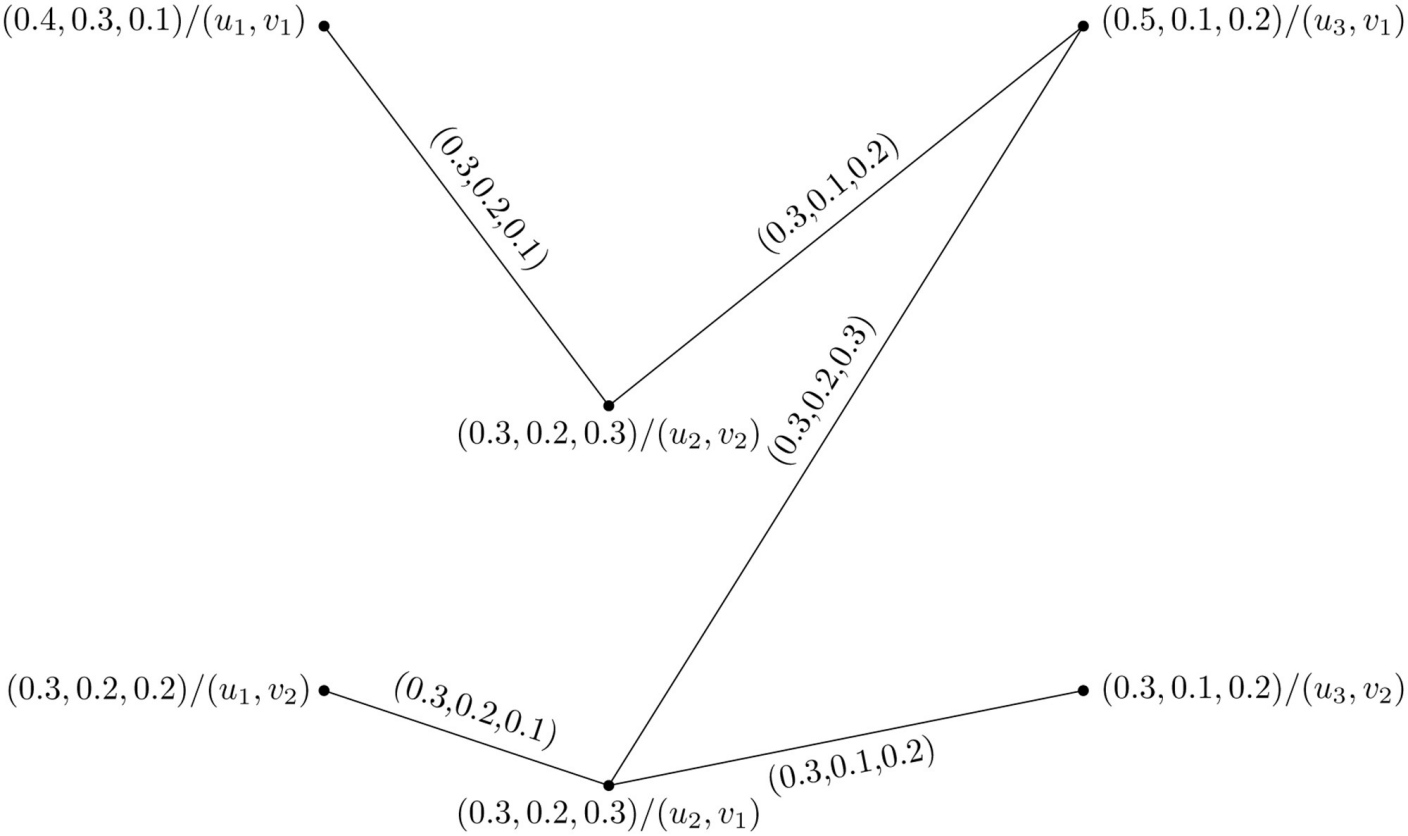
Direct product of picture fuzzy hypersoft graphs.

Consider two PFHSG $Y_{1}=\left(U_{1},W_{1}\right)$ and $Y_{2}=\left(U_{2},W_{2}\right)$ are shown in Figs 15 and 16. The direct product of $Y_{1}=\left(U_{1},W_{1}\right)$ and $Y_{2}=\left(U_{2},W_{2}\right)$ is shown in Fig 17.

**Definition 20: Direct Product of Picture Fuzzy Hypersoft Graphs**

Suppose ${Y_{1}}^{⋆}=\left(V_{1},E_{1}\right)$ and ${Y_{1}}^{⋆}=\left(V_{2},E_{2}\right)$ be two graph. The lexicographic product of $Y_{1}=\left(W_{1},U_{1}\right)$ and $Y_{2}=\left(W_{2},U_{2}\right)$ is denoted $Y_{1}⊙Y_{2}$ and defined by (W,U) where $W=({\overset{˘}{\mu }}_{W},{\overset{˘}{\eta }}_{W},{\overset{˘}{\nu }}_{W})$ and $U=({\overset{˘}{\mu }}_{U},{\overset{˘}{\eta }}_{U},{\overset{˘}{\nu }}_{U})$ are two PFS on $V=V_{1}\times V_{2}$, and $E=\left\{\left(l,l_{2}\right),\left(l,m_{2}\right)|\;l\in V_{1},\;l_{2}m_{2}\in E_{2}\right\}\cup \left\{\left(l_{1},n\right),\left(m_{1},n\right)|n\in V_{2},\;l_{1}m_{1}\in E_{1}\right\}$ respectively which satisfy the following conditions:

(i) For all $\left(l_{1},l_{2}\right)\in V_{1}\times V_{2}$
  (a) $\;\;{\overset{˘}{\mu }}_{W}\left(l_{1},l_{2}\right)={\overset{˘}{\mu }}_{W_{1}}\left(l_{1}\right)\vee {\overset{˘}{\mu }}_{W_{1}}\left(l_{2}\right)={\overset{˘}{\eta }}_{W_{1}}\left(l_{1}\right)\vee {\overset{˘}{\eta }}_{W_{1}}\left(l_{2}\right)={\overset{˘}{\nu }}_{W_{1}}\left(l_{1}\right)\wedge {\overset{˘}{\nu }}_{W_{1}}\left(l_{2}\right)$
(ii) For all $l\in V_{1}$ and $\left(l_{2},m_{2}\right)\in E_{2}$
  (a) ${\overset{˘}{\mu }}_{U}\left(\left(l,l_{2}\right)\left(l,m_{2}\right)\right)={\overset{˘}{\mu }}_{W_{1}}\left(l\right)\vee {\overset{˘}{\mu }}_{U_{2}}\left(l_{2}m_{2}\right)$
  (b) ${\overset{˘}{\eta }}_{U}\left(\left(l,l_{2}\right)\left(l,m_{2}\right)\right)={\overset{˘}{\eta }}_{W_{1}}\left(l\right)\vee {\overset{˘}{\eta }}_{U_{2}}\left(l_{2}m_{2}\right)$
  (c) ${\overset{˘}{\nu }}_{U}\left(\left(l,l_{2}\right)\left(l,m_{2}\right)\right)={\overset{˘}{\nu }}_{W_{1}}\left(l\right)\wedge {\overset{˘}{\nu }}_{U_{2}}\left(l_{2}m_{2}\right)$
(iii) For all $\left(l_{1}m_{1}\right)\in E_{1}$ and $\left(l_{2}m_{2}\right)\in E_{2}$
  (a) ${\overset{˘}{\mu }}_{U}\left(\left(l_{1},l_{2}\right)\left(m_{1},m_{2}\right)\right)={\overset{˘}{\mu }}_{U_{1}}\left(l_{1}m_{1}\right)\vee {\overset{˘}{\mu }}_{U_{2}}\left(l_{2}m_{2}\right)$
  (b) ${\overset{˘}{\eta }}_{U}\left(\left(l_{1},l_{2}\right)\left(m_{1},m_{2}\right)\right)={\overset{˘}{\eta }}_{U_{1}}\left(l_{1}m_{1}\right)\vee {\overset{˘}{\eta }}_{U_{2}}\left(l_{2}m_{2}\right)$
  (c) ${\overset{˘}{\nu }}_{U}\left(\left(l_{1},l_{2}\right)\left(m_{1},m_{2}\right)\right)={\overset{˘}{\nu }}_{U_{1}}\left(l_{1}m_{1}\right)\wedge {\overset{˘}{\nu }}_{U_{2}}\left(l_{2}m_{2}\right)$

**Definition 21: Strong Product of Picture Fuzzy Hypersoft Graphs**

Suppose ${Y_{1}}^{⋆}=\left(V_{1},E_{1}\right)$ and ${Y_{1}}^{⋆}=\left(V_{2},E_{2}\right)$ be two graph. The strong product of $Y_{1}=\left(W_{1},U_{1}\right)$ and $Y_{2}=\left(W_{2},U_{2}\right)$ is denoted ${\left(Y_{1}Y_{2}\right)}^{•}$ and defined by (W,U) where $W=({\overset{˘}{\mu }}_{W},{\overset{˘}{\eta }}_{W},{\overset{˘}{\nu }}_{W})$ and $U=({\overset{˘}{\mu }}_{U},{\overset{˘}{\eta }}_{U},{\overset{˘}{\nu }}_{U})$ are two PFS on $V=V_{1}\times V_{2}$, and $E=\left\{\left(l,l_{2}\right),\left(l,m_{2}\right)|\;l\in V_{1},\;l_{2}m_{2}\in E_{2}\right\}\cup \left\{\left(l_{1},n\right),\left(m_{1},n\right)|n\in V_{2},\;l_{1}m_{1}\in E_{1}\right\}\cup \left\{\left(l_{1},l_{2}\right),\left(m_{1},m_{2}\right)|\;l_{2}m_{2}\in V_{2}\;l_{2}\neq m_{2},\;l_{1}m_{1}\in E_{1}\right\}$ respectively which satisfy the following conditions:

(i) For all $\left(l_{1},l_{2}\right)\in V_{1}\times V_{2}$
  (a) $\;\;{\overset{˘}{\mu }}_{W}\left(l_{1},l_{2}\right)={\overset{˘}{\mu }}_{W_{1}}\left(l_{1}\right)\vee {\overset{˘}{\mu }}_{W_{1}}\left(l_{2}\right)$
  (b) $\;\;{\overset{˘}{\eta }}_{W}\left(l_{1},l_{2}\right)={\overset{˘}{\eta }}_{W_{1}}\left(l_{1}\right)\vee {\overset{˘}{\eta }}_{W_{1}}\left(l_{2}\right)$
  (c) $\;\;{\overset{˘}{\nu }}_{W}\left(l_{1},l_{2}\right)={\overset{˘}{\nu }}_{W_{1}}\left(l_{1}\right)\wedge {\overset{˘}{\nu }}_{W_{1}}\left(l_{2}\right)$
(ii) For all $l\in V_{1}$ and $\left(l_{2}m_{2}\right)\in E_{2}$
  (a) ${\overset{˘}{\mu }}_{U}\left(\left(l_{1},l_{2}\right)\left(l_{1},m_{2}\right)\right)={\overset{˘}{\mu }}_{W_{1}}\left(l_{1}\right)\vee {\overset{˘}{\mu }}_{U_{2}}\left(l_{2}m_{2}\right)$
  (b) ${\overset{˘}{\eta }}_{U}\left(\left(l_{1},l_{2}\right)\left(l_{1},m_{2}\right)\right)={\overset{˘}{\eta }}_{W_{1}}\left(l_{1}\right)\vee {\overset{˘}{\eta }}_{U_{2}}\left(l_{2}m_{2}\right)$
  (c) ${\overset{˘}{\nu }}_{U}\left(\left(l_{1},l_{2}\right)\left(l_{1},m_{2}\right)\right)={\overset{˘}{\nu }}_{W_{1}}\left(l_{1}\right)\wedge {\overset{˘}{\nu }}_{U_{2}}\left(l_{2}m_{2}\right)$
(iii) For all $l_{1}m_{1}\in E_{1}$ and $\left(l_{2},m_{2}\right)\in E_{2}$
  (a) ${\overset{˘}{\mu }}_{U}\left(\left(l_{1},l_{2}\right)\left(l_{1},m_{2}\right)\right)={\overset{˘}{\mu }}_{W_{2}}\left(l_{2}m_{2}\right)\vee {\overset{˘}{\mu }}_{U_{1}}\left(l_{1}m_{1}\right)$
  (b) ${\overset{˘}{\eta }}_{U}\left(\left(l_{1},l_{2}\right)\left(l_{1},m_{2}\right)\right)={\overset{˘}{\eta }}_{W_{2}}\left(l_{2}m_{2}\right)\vee {\overset{˘}{\eta }}_{U_{1}}\left(l_{1}m_{1}\right)$
  (c) ${\overset{˘}{\nu }}_{U}\left(\left(l_{1},l_{2}\right)\left(l_{1},m_{2}\right)\right)={\overset{˘}{\nu }}_{W_{2}}\left(l_{2}m_{2}\right)\wedge {\overset{˘}{\nu }}_{U_{1}}\left(l_{1}m_{1}\right)$
(iv) For all $n\in V_{2}$ and $\left(l_{1},m_{1}\right)\in E_{1}$
  (a) ${\overset{˘}{\mu }}_{U}\left(\left(l_{1},n\right)\left(m_{1},n\right)\right)={\overset{˘}{\mu }}_{W_{2}}\left(n\right)\vee {\overset{˘}{\mu }}_{U_{1}}\left(l_{1}m_{1}\right)$
  (b) ${\overset{˘}{\eta }}_{U}\left(\left(l_{1},n\right)\left(m_{1},n\right)\right)={\overset{˘}{\eta }}_{W_{2}}\left(n\right)\vee {\overset{˘}{\eta }}_{U_{1}}\left(l_{1}m_{1}\right)$
  (c) ${\overset{˘}{\nu }}_{U}\left(\left(l_{1},n\right)\left(m_{1},n\right)\right)={\overset{˘}{\nu }}_{W_{2}}\left(n\right)\wedge {\overset{˘}{\nu }}_{U_{1}}\left(l_{1}m_{1}\right)$

## 6 Application of picture fuzzy hypersoft graphs in decision support analysis

PFHSS is a vital hybrid structure to deal with problems faced in the real world. Studying inconsistent, incomplete, indeterminate, and multi-argument facts is highly beneficial when discussing problems in medical science, engineering, and business studies. Due to its versatile nature and wide range of applications, PFHSS has become an interesting and inventive subject for researchers. So, PFHSG can provide solutions to these kinds of problems. Here, the PFHS graph is used to deal with MADMP, and an algorithm is proposed to outline the thought process that illustrates the model’s working.

**Algorithm**:

Step 1: Calculate the impact coefficient between the attributes K by ${\$}_{ij}=\sqrt{\frac{{\overset{˘}{\mu }}_{W_{ij}}+{\overset{˘}{\eta }}_{W_{ij}}+{\overset{˘}{\nu }}_{W_{ij}}}{3}}$, ${\$}_{ij}=\left\{{\overset{˘}{\mu }}_{W_{ij}},{\overset{˘}{\eta }}_{W_{ij}},{\overset{˘}{\nu }}_{W_{ij}}\right\}$ is the PFHS graph edge between nodes K for i, j = 1, 2, …, n, and ${\$}_{ij}={\$}_{ji}$.

Step 2: Find the attribute of the alternative ${\overset{˘}{s}}_{i}$ below: ${\overset{˘}{s}}_{i}=\left({\overset{ˇ}{\mu }}_{W_{i}},{\overset{ˇ}{\eta }}_{W_{i}},{\overset{ˇ}{\nu }}_{W_{i}}\right)=\frac{1}{3}\sum_{j=1}^{n}w_{i}\left(\sum_{p=1}^{n}{\$}_{pj}c_{ip}\right)$

Step 3: Computation of the score functions using the follwoing equation: score $\left({\overset{˘}{s}}_{i}\right)=\frac{1}{2}\left[1+{\overset{ˇ}{\mu }}_{W_{i}}-2{\overset{ˇ}{\eta }}_{W_{i}}-{\overset{ˇ}{\nu }}_{W_{i}}\right]$.

Step 4: Selection of the best alternative by ranking the alternatives.

Step 5: Reporting of results.

## 7 Product risk assessment based for optimal design of investment strategies

In this section, numerical examples for the PFHSG MADM problem with picture fuzzy and hypersoft information are used to present the application of the proposed algorithms. An owner of supermarket wants to invest money and buy a variety of products based on numerous factors illustrated in the figure below. Now, a smart investment would be to to buy the products that have highest optimal value in terms of Return on investment, environmental factors, and shelf life of the product etc. Based on these factors, we propose an algorithm based on PFHS graphical structure to find the risk in investment when buying each product while addressing each of the factors addressed in the figure. The problem is illustrated below:

Suppose $\overset{˘}{S}=\{\overset{˘}{s_{1}}=\text{Bakery}$, $\overset{˘}{s_{2}}=\text{Butcher}\;\text{shop}$, $\overset{˘}{s_{3}}=\text{Dairy}\;\text{store}$, $\overset{˘}{s_{4}}=\text{Pharmacy}$} are four measurable alternatives for a businessperson starting a new business. Let *E* = {*a*_1_, *a*_2_, *a*_3_, *a*_4_} be attributes set where each *a*_*i*_ stands for Gross margin, Product monthly sales, Customer satisfactions and environmental impact analysis respectively, whose corresponding attribute values are {*H*_1_, *H*_2_, *H*_3_} respectively. In order for the businessperson to efficiently invest in these four alternative investment options, a risk analysis must be done that addresses all the factors listed below to generate the quickest return on investment while also considering factors like the market image of the store and quality of the products.

Let the factors being considered for the risk analysis assessment be:

- *H*_1_ = {*b*_11_ = Product price, *b*_12_ = Labor cost, *b*_13_ = Inventory methods}
- *H*_2_ = {*b*_21_ = Brand reputation, *b*_22_ = Sale price, *b*_23_ = Customer necessity in daily life}
- *H*_3_ = {*b*_31_ = Customer services, *b*_32_ = Product return policy, *b*_33_ = Overall flexibility when buying particular product}
- *H*_4_ = {*b*_41_ = Seasonal effect on product, *b*_42_ = Effect of pollution, *b*_43_ = Product spoilage time, *b*_44_ = Accidental loss}

Now,

$$\tilde{L}=H_{1}\times H_{2}\times H_{3}\times H_{4}$$

There are one hundred and eight outcomes but for simplicity, only three outcomes are explored and risk assessment is done on these three outcomes. The edge set of the outcomes is presented in Table 3.

$$\tilde{L}=\{\begin{array}{c} \;{\overset{˘}{t}}_{1}=\left(b_{11},b_{22},b_{31},b_{41}\right),\;\;\;{\overset{˘}{t}}_{2}=\left(b_{12},b_{23},b_{33},b_{41}\right),\\ {\overset{˘}{t}}_{3}=\left(b_{13},b_{22},b_{33},b_{43}\right) \end{array}\}$$

$$\left(\overset{˘}{H},\tilde{L}\right)=\{\begin{array}{c} \overset{˘}{H}\left({\overset{˘}{t}}_{1}\right)=\{\langle 0.5,0.1,0.1\rangle /{\overset{˘}{s}}_{1},\langle 0.3,0.2,0.2\rangle /{\overset{˘}{s}}_{2},\\ \;\;\;\;\;\;\;\;\;\;\;\;\langle 0.3,0.2,0.3\rangle /{\overset{˘}{s}}_{3},\langle 0.5,0.1,0.2\rangle /{\overset{˘}{s}}_{4}\},\\ \overset{˘}{H}\left({\overset{˘}{t}}_{2}\right)=\{\langle 0.5,0.1,0.1\rangle /{\overset{˘}{s}}_{1},\langle 0.3,0.1,0.2\rangle /{\overset{˘}{s}}_{2},\\ \;\;\;\;\;\;\;\;\;\;\;\;\langle 0.8,0.1,0.1\rangle /{\overset{˘}{s}}_{3},\langle 0.4,0.1,0.2\rangle /{\overset{˘}{s}}_{4},\},\\ \overset{˘}{H}\left({\overset{˘}{t}}_{3}\right)=\{\langle 0.3,0.2,0.1\rangle /{\overset{˘}{s}}_{1},\langle 0.1,0.1,0.6\rangle /{\overset{˘}{s}}_{2},\\ \;\;\;\;\;\;\;\;\;\;\;\;\langle 0.2,0.1,0.7\rangle /{\overset{˘}{s}}_{3},\langle 0.4,0.1,0.1\rangle /{\overset{˘}{s}}_{4}\} \end{array}\}$$

is picture fuzzy hypersoft set.

**Table 3:** Vertices set of PFHSG.

U	${\overset{˘}{t}}_{1}$	${\overset{˘}{t}}_{2}$	${\overset{˘}{t}}_{3}$
${\overset{˘}{s}}_{1}$	〈0.5, 0.1, 0.1〉	〈0.5, 0.1, 0.1〉	〈0.3, 0.2, 0.1〉
${\overset{˘}{s}}_{2}$	〈0.3, 0.2, 0.2〉	〈0.3, 0.1, 0.2〉	〈0.1, 0.1, 0.6〉
${\overset{˘}{s}}_{3}$	〈0.3, 0.2, 0.3〉	〈0.8, 0.1, 0.1〉	〈0.2, 0.1, 0.7〉
${\overset{˘}{s}}_{4}$	〈0.5, 0.1, 0.2〉	〈0.4, 0.1, 0.2〉	〈0.4, 0.1, 0.1〉

Let M = (*c*_*ij*_)_4×3_ be a picture fuzzy hypersoft decision matrix:

$$M=(\begin{array}{ccc} \left\langle 0.5,0.1,0.1\right\rangle & \left\langle 0.5,0.1,0.1\right\rangle & \left\langle 0.3,0.2,0.1\right\rangle \\ \left\langle 0.3,0.2,0.2\right\rangle & \left\langle 0.3,0.1,0.2\right\rangle & \left\langle 0.1,0.1,0.6\right\rangle \\ \left\langle 0.3,0.2,0.3\right\rangle & \left\langle 0.8,0.1,0.1\right\rangle & \left\langle 0.2,0.1,0.7\right\rangle \\ \left\langle 0.5,0.1,0.2\right\rangle & \left\langle 0.4,0.1,0.2\right\rangle & \left\langle 0.4,0.1,0.1\right\rangle \end{array})$$

**Fig 18:**
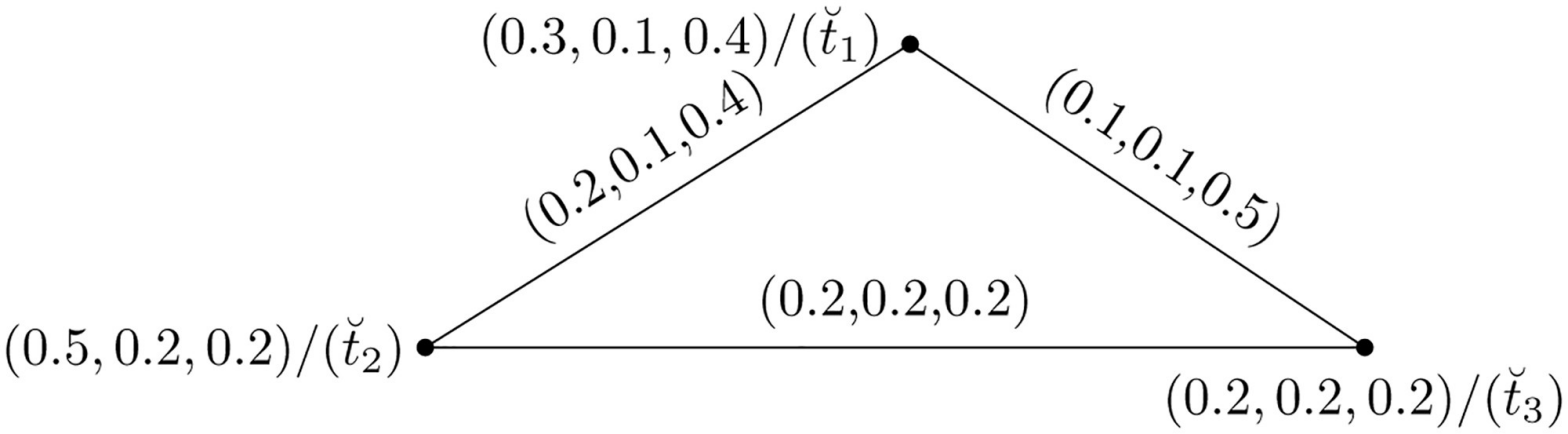
Picture fuzzy hypersoft graphs.

Also, a complete graph C=(K,D) represents a relationship between attribute values $\left\{{\overset{˘}{t}}_{1},{\overset{˘}{t}}_{2},{\overset{˘}{t}}_{3}\right\}$ shown in Fig 18, where $K=\{{\overset{˘}{t}}_{1},{\overset{˘}{t}}_{2},{\overset{˘}{t}}_{3}\}$ and $D=\{e_{12}=\left({\overset{˘}{t}}_{1},{\overset{˘}{t}}_{2}\right),e_{13}=\left({\overset{˘}{t}}_{1},{\overset{˘}{t}}_{3}\right),e_{23}=\left({\overset{˘}{t}}_{2},{\overset{˘}{t}}_{3}\right)\}$ are the vertices and edge sets, respectively. The sets are as follows: $\{{\overset{˘}{t}}_{1}=\langle 0.3,0.1,0.4\rangle$, ${\overset{˘}{t}}_{2}=\langle 0.5,0.2,0.2\rangle$, ${\overset{˘}{t}}_{3}=\langle 0.2,0.2,0.2\rangle \}$ and {*e*_12_ = 〈0.2, 0.1, 0.4〉, *e*_13_ = 〈0.1, 0.1, 0.5〉, *e*_23_ = 〈0.2, 0.2, 0.2〉}.

The weight vector W of $\tilde{L}$ is given by $W=\left(w_{1},w_{2},w_{3}\right)=\left(0.38,0.32,0.30\right)$.

Step 1: Computation of the impact coefficient between the attributes K:

${\$}_{12}=\sqrt{\frac{{\overset{˘}{\mu }}_{W_{12}}+{\overset{˘}{\eta }}_{W_{12}}+{\overset{˘}{\nu }}_{W_{12}}}{3}}=\sqrt{\frac{0.2+0.1+0.4}{3}}=\sqrt{0.233}=0.483$

${\$}_{13}=\sqrt{\frac{{\overset{˘}{\mu }}_{W_{13}}+{\overset{˘}{\eta }}_{W_{13}}+{\overset{˘}{\nu }}_{W_{13}}}{3}}=\sqrt{\frac{0.1+0.1+0.5}{3}}=\sqrt{0.233}=0.483$

${\$}_{23}=\sqrt{\frac{{\overset{˘}{\mu }}_{W_{23}}+{\overset{˘}{\eta }}_{W_{23}}+{\overset{˘}{\nu }}_{W_{23}}}{3}}=\sqrt{\frac{0.2+0.2+0.2}{3}}=\sqrt{0.2}=0.447$

${\$}_{11}=\sqrt{\frac{{\overset{˘}{\mu }}_{W_{11}}+{\overset{˘}{\eta }}_{W_{11}}+{\overset{˘}{\nu }}_{W_{11}}}{3}}=\sqrt{\frac{1.0+0.0+0.0}{3}}=\sqrt{0.333}=0.577$

Step 2: The attributes of the alternative ${\overset{˘}{s}}_{i}$ is calculated below:

$${\overset{˘}{s}}_{1}=\frac{1}{3}\left[w_{1}\left({\$}_{11}c_{11}+{\$}_{21}c_{12}+{\$}_{31}c_{13}\right)+w_{2}\left({\$}_{12}c_{11}+{\$}_{22}c_{12}+{\$}_{31}c_{13}\right)+w_{3}\left({\$}_{13}c_{11}+{\$}_{23}c_{12}+{\$}_{33}c_{13}\right)\right]=\frac{1}{3}\left[0.38\left(\left(0.577\right)\langle 0.5,0.1,0.1\rangle +\left(0.483\right)\langle 0.5,0.1,0.1\rangle +\left(0.483\right)\langle 0.3,0.2,0.1\rangle \right)+0.32\left(\left(0.483\right)\langle 0.5,0.1,0.1\rangle +\left(0.577\right)\langle 0.5,0.1,0.1\rangle +\left(0.483\right)\langle 0.3,0.2,0.1\rangle \right)+0.30\left(\left(0.483\right)\langle 0.5,0.1,0.1\rangle +\left(0.447\right)\langle 0.5,0.1,0.1\rangle +\left(0.577\right)\langle 0.3,0.2,0.1\rangle \right)\right]=\left(0.200,0.068,0.051\right)$$

$${\overset{˘}{s}}_{2}=\frac{1}{3}\left[w_{1}\left({\$}_{11}c_{21}+{\$}_{21}c_{22}+{\$}_{31}c_{23}\right)+w_{2}\left({\$}_{12}c_{21}+{\$}_{22}c_{22}+{\$}_{31}c_{23}\right)\right)+w_{3}\left({\$}_{13}c_{21}+{\$}_{23}c_{22}+{\$}_{33}c_{23}\right)]=\frac{1}{3}\left[0.38\left(\left(0.577\right)\langle 0.3,0.2,0.2\rangle +\left(0.483\right)\langle 0.3,0.1,0.2\rangle +\left(0.483\right)\langle 0.1,0.1,0.6\rangle \right)+0.32\left(\left(0.483\right)\langle 0.3,0.2,0.2\rangle +\left(0.577\right)\langle 0.3,0.1,0.2\rangle +\left(0.483\right)\langle 0.1,0.1,0.6\rangle \right)+0.30\left(\left(0.483\right)\langle 0.3,0.2,0.2\rangle +\left(0.447\right)\langle 0.3,0.1,0.2\rangle +\left(0.577\right)\langle 0.1,0.1,0.6\rangle \right)\right]=\left(0.119,0.085,0.153\right)$$

$${\overset{˘}{s}}_{3}=\frac{1}{3}\left[w_{1}\left({\$}_{11}c_{31}+{\$}_{21}c_{32}+{\$}_{31}c_{33}\right)+w_{2}\left({\$}_{12}c_{31}+{\$}_{22}c_{32}+{\$}_{31}c_{33}\right)\right)+w_{3}\left({\$}_{13}c_{31}+{\$}_{23}c_{32}+{\$}_{33}c_{33}\right)]=\frac{1}{3}\left[0.38\left(\left(0.577\right)\langle 0.3,0.2,0.3\rangle +\left(0.483\right)\langle 0.8,0.1,0.1\rangle +\left(0.483\right)\langle 0.2,0.1,0.7\rangle \right)+0.32\left(\left(0.483\right)\langle 0.3,0.2,0.3\rangle +\left(0.577\right)\langle 0.8,0.1,0.1\rangle +\left(0.483\right)\langle 0.2,0.1,0.7\rangle \right)+0.30\left(\left(0.483\right)\langle 0.30.2,0.3\rangle +\left(0.447\right)\langle 0.8,0.1,0.1\rangle +\left(0.577\right)\langle 0.2,0.1,0.7\rangle \right)\right]=\left(0.223,0.068,0.187\right)$$

$${\overset{˘}{s}}_{4}=\frac{1}{3}\left[w_{1}\left({\$}_{11}c_{41}+{\$}_{21}c_{42}+{\$}_{31}c_{43}\right)+w_{2}\left({\$}_{12}c_{41}+{\$}_{22}c_{42}+{\$}_{31}c_{42}\right)\right)+w_{3}\left({\$}_{13}c_{41}+{\$}_{23}c_{42}+{\$}_{33}c_{43}\right)]=\frac{1}{3}\left[0.38\left(\left(0.577\right)\langle 0.5,0.1,0.2\rangle +\left(0.483\right)\langle 0.4,0.1,0.2\rangle +\left(0.483\right)\langle 0.4,0.1,0.1\rangle \right)+0.32\left(\left(0.483\right)\langle 0.5,0.1,0.2\rangle +\left(0.577\right)\langle 0.4,0.1,0.2\rangle +\left(0.483\right)\langle 0.4,0.1,0.1\rangle \right)+0.30\left(\left(0.483\right)\langle 0.5,0.1,0.2\rangle +\left(0.447\right)\langle 0.4,0.1,0.1\rangle +\left(0.577\right)\langle 0.4,0.1,0.2\rangle \right)\right]=\left(0.221,0.051,0.085\right)$$

Step 3: Computation of Final score functions for ranking:

$$\text{score}\left({\overset{˘}{s}}_{1}\right)=\frac{1}{2}\left[1+{\overset{ˇ}{\mu }}_{W_{1}}-2{\overset{ˇ}{\eta }}_{W_{1}}-{\overset{ˇ}{\nu }}_{W_{1}}\right]=\frac{1}{2}\left[1+0.200-2\left(0.068\right)-0.051\right]=0.438$$

$$\text{score}\left({\overset{˘}{s}}_{2}\right)=\frac{1}{2}\left[1+{\overset{ˇ}{\mu }}_{W_{2}}-2{\overset{ˇ}{\eta }}_{W_{2}}-{\overset{ˇ}{\nu }}_{W_{2}}\right]=\frac{1}{2}\left[1+0.200-2\left(0.068\right)-0.051\right]=0.398$$

$$\text{score}\left({\overset{˘}{s}}_{3}\right)=\frac{1}{2}\left[1+{\overset{ˇ}{\mu }}_{W_{3}}-2{\overset{ˇ}{\eta }}_{W_{3}}-{\overset{ˇ}{\nu }}_{W_{3}}\right]=\frac{1}{2}\left[1+0.200-2\left(0.068\right)-0.051\right]=0.450$$

$$\text{score}\left({\overset{˘}{s}}_{4}\right)=\frac{1}{2}\left[1+{\overset{ˇ}{\mu }}_{W_{4}}-2{\overset{ˇ}{\eta }}_{W_{4}}-{\overset{ˇ}{\nu }}_{W_{4}}\right]=\frac{1}{2}\left[1+0.200-2\left(0.068\right)-0.051\right]=0.517$$

**Fig 19:**
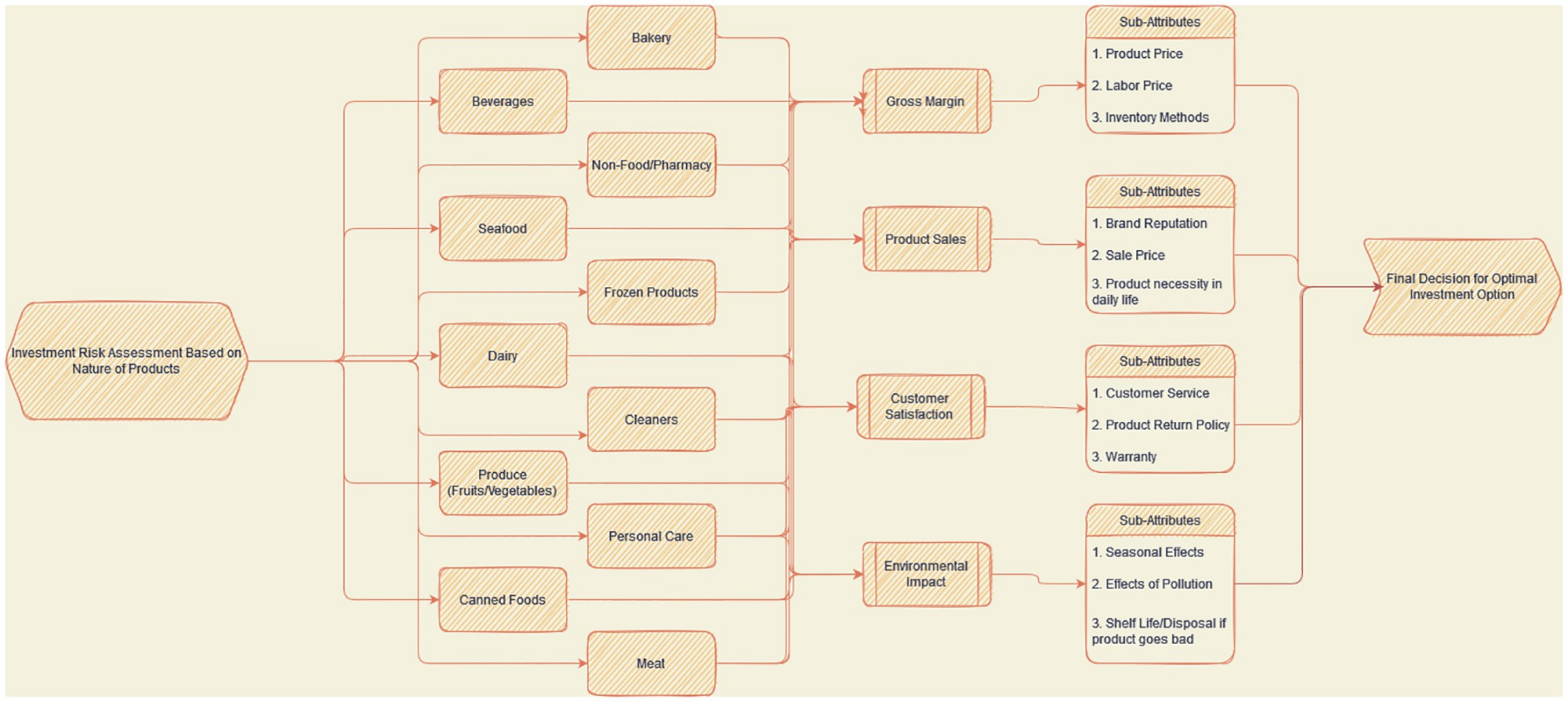
Factors considered for the risk assessment for supermarket products.

Hence $\left({\overset{˘}{s}}_{4}\right)$ is best choice because score $({\overset{˘}{s}}_{4})⪰$ score $({\overset{˘}{s}}_{3})⪰$ score $({\overset{˘}{s}}_{1})⪰$ score $\left({\overset{˘}{s}}_{2}\right)$. This provides a clear indication that the Pharmacy section of the supermarket is the best for investment as it has the lowest risk for loss of investment while considering the factors listed in Fig 19. Following the Pharmacy is the Dairy products section of the store, with the Bakery at the third position and the Meat section at the fourth. This example only describes a minimal example for an explanation but can address the sub-attributes of attributes in a graphical manner which are in most cases neglected (Other than Hypersoft Structures) due to the inability to address all the factors addressed in Table 4. This tool is great for obtaining a graphical depiction for development and solving a decision-making problem. It works great as a decision support system as it has potential applications in just about any field, from medical diagnostic systems to mechanical engineering problems.

**Table 4:** Comparison between PFHSG and some existing fuzzy graph theories.

Name	Year	Structure	*M*_*sp*_	*N*_*Msp*_	*N*_*trl*_	*P*_*rmtr*_	*A*_*trbtes*_	*S*_*trbts*_
Rosenfeld [2]	1975	Fuzzy Graph	Y	N	N	N	Y	N
Parvathi et al. [66]	2005	Intuitionistic Fuzzy Graph	Y	Y	N	N	Y	N
Zuo et al. [35]	2019	Picture Fuzzy Graph	Y	Y	Y	N	Y	N
Thumbakara et al. [67]	2014	Soft Graph	Y	N	N	Y	Y	N
Akram et al. [68]	2015	Fuzzy Soft Graph	Y	N	N	Y	Y	N
Shahzadi et al. [69]	2018	Intuitionistic Fuzzy Soft Graph	Y	Y	N	Y	Y	N
Chellamani et al. [70]	2021	Picture Fuzzy Soft Graph	Y	Y	Y	Y	Y	N
Saeed et al. [59]	2021	Hyper Soft Graph	Y	N	N	Y	Y	Y
Saeed et al. [15]	2021	Complex Fuzzy Hypersoft Graph	Y	N	N	Y	Y	Y
Yolcu et al. [71]	2021	Intuitionistic Fuzzy Hypersoft Graph	Y	Y	N	Y	Y	Y
Proposed Techniques	2022	Picture Fuzzy Hypersoft Graph	Y	Y	Y	Y	Y	Y

## 8 Comparative analysis

A comparative analysis of the proposed structure is provided in Table 4. Previously available structures in literature can address some attributes using fuzzy parameters like membership and non-membership functions, but they fall short when there are sub-attributes. These are then addressed using Hypersoft structures that can deal with such issues, and the proposed hybrid structure covers the whole spectrum of characteristics allowing for deep and thorough analysis.

## 9 Conclusion

Risk evaluation has always been of great interest for individuals wanting to invest in various businesses, especially in the marketing and product sale centres. A finely detailed evaluation of the risk factor can lead to better returns in terms of investment in a particular business. This presents a MADM problem requiring numerous factors to be addressed simultaneously. Graph theory is an essential tool for solving MADM problems in various kinds of hybrid structures like PFS, PFSS, HS, and PFHSS. Picture fuzzy hypersoft graph is a new concept in the theory of graphs. PFHS graph has been applied to solve problems containing consistent, inconsistent, and multi-argument information. This article introduces basic operations like union, composition, cartesian product, direct product, and joint of PFHS graph. We proposed an algorithm in which a relationship is created among attributes and alternatives in the decision process to obtain the desired result. The structure is then used to address a risk analysis problem for investment distribution for product buying in a supermarket. In the future, we can extend this structure in neutrosophic, spherical and T-spherical hypersoft sets while applying the structures to actual data for real-world applications.
